# Understanding the sugar beet holobiont for sustainable agriculture

**DOI:** 10.3389/fmicb.2023.1151052

**Published:** 2023-04-17

**Authors:** Adrian Wolfgang, Nora Temme, Ralf Tilcher, Gabriele Berg

**Affiliations:** ^1^Institute of Environmental Biotechnology, Graz University of Technology, Graz, Austria; ^2^KWS SAAT SE & Co. KGaA, Einbeck, Germany; ^3^Microbiome Biotechnology Department, Leibniz-Institute for Agricultural Engineering and Bioeconomy (ATB), Potsdam, Germany; ^4^Institute for Biochemistry and Biology, University of Potsdam, Potsdam, Germany

**Keywords:** biofertilization, *Beta vulgaris*, *Rhizoctonia*, phylosymbiosis, microbiome, biocontrol, soil-borne pathogens

## Abstract

The importance of crop-associated microbiomes for the health and field performance of plants has been demonstrated in the last decades. Sugar beet is the most important source of sucrose in temperate climates, and—as a root crop—yield heavily depends on genetics as well as on the soil and rhizosphere microbiomes. Bacteria, fungi, and archaea are found in all organs and life stages of the plant, and research on sugar beet microbiomes contributed to our understanding of the plant microbiome in general, especially of microbiome-based control strategies against phytopathogens. Attempts to make sugar beet cultivation more sustainable are increasing, raising the interest in biocontrol of plant pathogens and pests, biofertilization and –stimulation as well as microbiome-assisted breeding. This review first summarizes already achieved results on sugar beet-associated microbiomes and their unique traits, correlating to their physical, chemical, and biological peculiarities. Temporal and spatial microbiome dynamics during sugar beet ontogenesis are discussed, emphasizing the rhizosphere formation and highlighting knowledge gaps. Secondly, potential or already tested biocontrol agents and application strategies are discussed, providing an overview of how microbiome-based sugar beet farming could be performed in the future. Thus, this review is intended as a reference and baseline for further sugar beet-microbiome research, aiming to promote investigations in rhizosphere modulation-based biocontrol options.

## 1. Introduction

The holobiont concept (Zilber-Rosenberg and Rosenberg, 2008) changed the view on microbes in many scientific disciplines. It states that practically all multicellular lifeforms are inhabited, depending on—or at least are affected by—the interplay with microbial life. The collective genome of plant-associated microbiota exceeds the host genome in both size and number of functions by far and is thus referred to as its second genome (Berendsen et al., 2012; de la Fuente Cant et al., 2020). Given the importance of plant-associated microbes for the health, vigor, and resilience of their host, the microbiome of plants and its modulation is a potential key factor for crop management and crop development in the future (Berg et al., 2015, 2021; Mendes and Raaijmakers, 2015).

Sugar beet (*Beta vulgaris* ssp. *vulgaris*, L.) is the most important regional source of sucrose in moderate climates of the northern hemisphere. Its biomass production is ranked eighth amongst the most produced field crops worldwide (FAOSTAT, 2022). Sugar beets are biennial, meaning that flowers and seeds are produced in the second year. Since flowering detracts sucrose from taproots, sugar beets are harvested annually. The wild ancestor of all beet crops is the sea beet (*Beta maritima* L.), a native plant still frequently found on European coastlines. Sugar beet thrives on most soil types, as long as pH is near neutral, easing its geographically widespread cultivation (Draycott, 2003). In contrast to many other crops, the breeding of sugar beet out of the Silesian Beet happened in times when the basics of genetics were understood. Therefore, its development and breeding trends over the decades are comparably well documented (Panella and Lewellen, 2007). Early sugar beet cultivars were bred in Northern Europe, a region with a non-humid, temperate climate and low pest and disease pressure. When these cultivars were planted in other regions, the yield was severely decimated by pests and pathogens (Panella and Lewellen, 2007). Sugar beet was intensively studied regarding physiology, anatomy, chemical, biochemical constitution, genomic traits, nutrient requirements, and convenient agricultural practices to optimize yield in the last 150 years, and was first genome sequenced in 2014 (Dohm et al., 2014). Still, leaf pathogens, root and storage rots, and microbes interfering with sucrose extractions illustrate the importance of sugar beet-associated microbial communities for both plant health and yield. All these mentioned facts make sugar beet an interesting model plant for microbiome research.

Despite the widespread cultivation of sugar beet, our knowledge in sugar beet microbiomes and microbiome-based strategies in future agricultural systems have not reached their full potential thus far. To fully exploit this potential for crop protection and plant growth promotion (PGP), a deep and holistic understanding of both the plant itself and the environment-plant interactions is crucial. Since the rhizosphere is the primary soil-plant interface, we have to especially emphasize the establishment, formation, and dynamics of its microbiome in this context. We hereby try to connect current knowledge about sugar beet-associated microbial communities to their physical, chemical, and biological context, namely the specific traits of the host plant. We aim to describe the sugar beet holobiont as defined by Berg et al. (2020), as the entirety of the microbial community members and its “theater of activity”. In the first section of this review, we will provide an overview of the current knowledge on sugar beet microbiome to be considered in experimental setups of future studies, highlight knowledge gaps, and discuss the sugar beet holobiont following its ontology from seed to postharvest roots. The second section summarizes potential or already tested biocontrol agents and their natural occurrence in the plant host and presents the current application strategies for microbiome-based agricultural practices.

## 2. Part I: Microbial journeys on and in sugar beets: from seed to beet

### 2.1. Seed endophytes as source for rhizosphere microbes

Plant holobionts represent dynamic systems where the plant and microbiota influence each other and both develop over time. During their life cycle, starting with seed germination, plants provide several microhabitats (root: rhizosphere and root endosphere; leaves and stems: phyllosphere; flowers: anthosphere; fruits: carposphere; seeds: spermosphere) with very different physicochemical properties. Consequently, these plant microhabitats harbor specific microbiota connected by endophytic communities (Hardoim et al., 2015), that may differ on very small spatial scales (Ottesen et al., 2013; Wassermann et al., 2019b; Kusstatscher et al., 2020). Endophytes may either migrate through the vascular system of plants (James et al., 2002; Compant et al., 2005, 2021) or move through the apoplast (Gasser et al., 2011; Compant et al., 2021), the latter requiring cell wall-degrading enzymes (Dong et al., 1994; James et al., 2002; Liu et al., 2017). In homologous organs, endophytic microbiomes partially differ according to phylogeny (Abdelfattah et al., 2021), even up to cultivar-specific differences in seeds (Rybakova et al., 2017; Wolfgang et al., 2020).

Seed tissues usually undergo serious physiological changes during maturation, including enrichment of starch and dehydration. Therefore, seed endophytes often require additional characteristics and adaptions, e.g., tolerance toward high osmotic pressure, amongst other typical traits (Truyens et al., 2015). Seed endophytes are difficult to discover because the majority are in a dormant, non-cultivable stage. Using microbial community fingerprinting methods developed in the 1990's, mainly based on PCRs amplifying 16Sr DNA genes, seed microbiota could be analyzed and were found to be surprisingly diverse (Berg and Raaijmakers, 2018). Nevertheless, the origin of seed endophytes is still a matter of debate, since it appears that plant taxa differ in to what extent endophytes are either horizontally acquired or vertically transmitted. Vertically transmitted seed endophytes represent a core community in seeds (Truyens et al., 2015; Nelson, 2018; Shahzad et al., 2018). However, seed endo- and ectophytes play an important role in early plant development, including rhizosphere development (Berg and Raaijmakers, 2018).

In sugar beet, an influence of both the genotype and the environment on microbial seed communities was reported, although a core community is conserved to some extent (Wolfgang et al., 2020). This microbial inheritance was equally observed in other crops, e.g., *Arabidopsis* (Truyens et al., 2013), maize (Johnston-Monje et al., 2016), oak (Abdelfattah et al., 2021; Fort et al., 2021), beans (Klaedtke et al., 2016) and tomato (Bergna et al., 2018). Microbial inheritance represents the basis of the phylosymbiosis concept (Lim and Bordenstein, 2020). Dent et al. (2004) were the first to use culture-independent PCR-DGGE for seed community profiling in sugar beet. They discovered both bacterial and fungal DNA in the seed coat (=fruit) that showed specific band patterns depending on seed acreage, which was correlated with differing germination rates under field conditions. Bacterial seed communities appeared to be complex, while fungal communities seemed to be species-poor (Dent et al., 2004; Spanner et al., 2021). A commonality in bacterial sugar beet seed communities with other plant species is the high abundance of *Pseudomonas* and *Pantoea* (Truyens et al., 2015; Nelson, 2018; Wassermann et al., 2019a). Other genera contribute to the bacterial seed community to various extents, including *Paenibacillus, Sphingomonas, Curtobacterium, Massilia, Methylobacterium, Saccharibacillus*, and *Kosakonia* (Wolfgang et al., 2020). Some fungal taxa known for phytopathogenic traits, e.g., *Cercospora, Fusarium*, and *Alternaria*, can establish in seeds *via* the xylem sap flow (Spanner et al., 2021), with negative implications for the next sugar beet generation. *Archaea* represent 0–1.1% relative abundance and mainly comprise *Woesearchaeia*, indicating a minor role of *Archaea* in seeds (Wolfgang et al., 2020). In addition to genetic and epigenetic information of the plant itself, seed endophyte communities can thus be interpreted as a third layer of inheritable information about the direct environment of the host plant, which is directly or indirectly provided and modified by the parental plant to the next generation of plants. This further indicates that the sugar beet seed community is modifiable by the substrate of the mother plant, which may have implications for sugar beet breeding or plant pathogen control.

**Figure 1:**
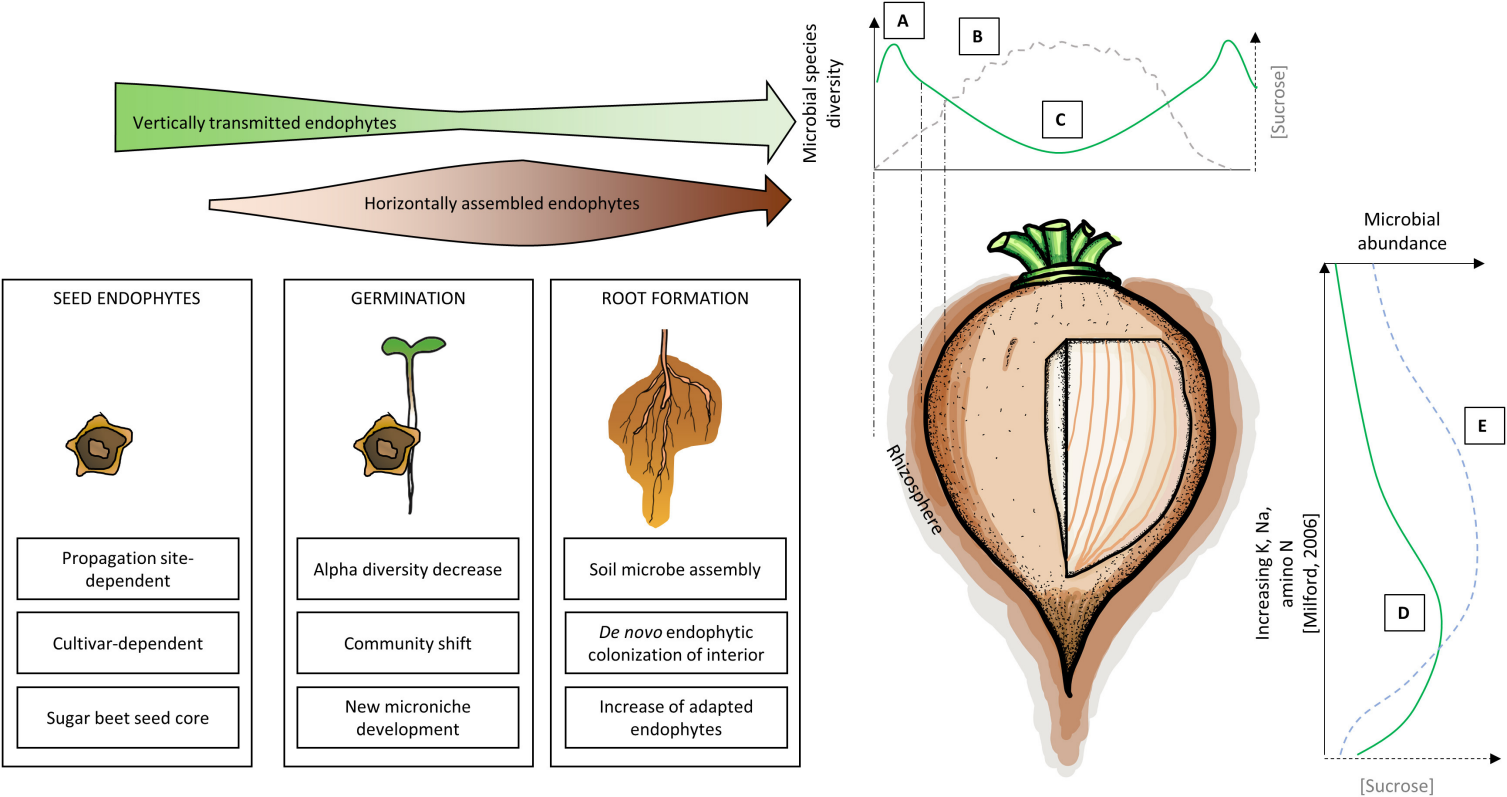
Simplified temporal **(Left)** and spatial **(Right)** holobiont model of sugar beet taproot. The arrow width indicates the relative importance of vertically and horizontally assembled endophytes **(Top Left)**. A: Root exudation and/or endophyte release leads to an increase in measured diversity in taproot-associated rhizosphere communities (Zachow et al., 2014; Cardinale et al., 2020; Wolfgang et al., 2020). CFU number in the peel can exceed the CFU number in the rhizosphere (Okazaki et al., 2014). B: Relative sugar content increases toward the center, higher in proximity to vascular bundles. Sucrose further decreases with increasing distance to the secondary cambia (Milford, 2006; Hoffmann and Kenter, 2018). C: Diversity decreases toward the center, while the relative abundance of copiotrophic bacteria increases (Lilley et al., 1996; Okazaki et al., 2014). D: Microbial abundance is highest in the root elongation zone near the root tip, with a high relative abundance of exudate responders, e.g., *Variovorax* and *Pseudomonas* (Jacobs et al., 1985; Lübeck et al., 2000; Shi et al., 2009a). E: The sugar content of beet tissue is highest in lower taproot (Milford, 2006).

The process of germination in sugar beet is epigeal (Milford, 2006). Germination is generally considered the most vulnerable life stage, where plants are highly susceptible to abiotic and biotic stressors (Hegarty, 1977). Interaction dynamics between seedling, seed-associated microbiomes, and soil microbiomes during seed germination, including the establishment and development of rhizosphere communities, may constitute the most decisive life stage for further plant health (Nelson, 2018). Dominating taxa in seeds also dominate in soilless germinated sugar beet seedling roots, namely *Kosakonia, Methylobacterium, Pantoea*, and *Pseudomonas* (Wolfgang et al., 2020). According to the mentioned study, ~63–83% of seed endophytes survive germination and colonize developing roots. *In vitro* roots show lower alpha diversity, a community shift, and a less balanced microbiome composition than seeds (Wolfgang et al., 2020), an effect as well observed in *Brassica* plants (Barret et al., 2015), maize (Johnston-Monje et al., 2016) and wheat (Huang et al., 2016). This indicates a first selection of vertically transmitted endophytes in germinating roots, although it is yet unassessed whether this process is stochastic or specific. However, the instability of the seedling community indicates a “reshuffling” of the seed community, potentially creating new niches for soil-derived microbes (Figure 1A). In this life stage, seed endophytes have several advantages compared to soil-derived microbiota to further colonize the seedling. Firstly, colonization of the newly germinated seedlings is more rapid, since they already inhabit the seed and do not have to compete for niches and nutrients yet. Secondly, they are already adapted to live inside plant tissue, and thirdly, they are protected to an extent from environmental stressors by host tissue (Hallmann and Berg, 2007; Kaga et al., 2009; Hardoim et al., 2012). Altogether, seed microbes provide ideal starter cultures for seedlings, while microorganisms acquired from soil allow an adaptation to local conditions (Bergna et al., 2018); both sources ensure an adapted microbiota assembly for the whole plant life cycle.

### 2.2. Microbial assembly and dynamics in the sugar beet rhizosphere

The rhizosphere is the critical soil-plant interface for resource exchange and the interplay between the plant host and soil microbiota (Weller et al., 2002; Philippot et al., 2013; Pantigoso et al., 2022). In the sugar beet rhizosphere, the number of cultivable bacteria and fungi is approximately 10^8^ and 2*10^5^ CFUs per gram fresh weight, respectively (Zachow et al., 2008). *Proteobacteria, Acidobacteria*, and *Actinobacteria* dominate the root community of 14d-old sugar beet cultivars. The archaeal family *Nitrososphaeraceae* is steadily represented but derived from soil (Wolfgang et al., 2020). Protists play a pivotal role in shaping sugar beet rhizosphere microbiomes (Bazany et al., 2022), but are rarely investigated on metagenomic levels. Interestingly, sugar beet rhizospheres were repeatedly reported to display a comparable (Mendes et al., 2011), or even higher bacterial alpha diversity than surrounding bulk soil in the early seedling stages (Zachow et al., 2014; Wolfgang et al., 2020), but also in the rhizosphere of pre-harvest taproots (Cardinale et al., 2020), while also significant lower rhizosphere alpha diversity compared to bulk soil was reported (Cui et al., 2022). Rhizosphere communities in vascular plants are usually less diverse than soil communities (Berendsen et al., 2012). This so-called “rhizosphere effect” is caused by specific root exudates that enrich or repel a specific fraction of the given soil community (Houlden et al., 2008; Berg and Smalla, 2009; de la Fuente Cant et al., 2020). There are several non-exclusive possible explanations for this deviant phenomenon in sugar beet that can be categorized into methodological and ecological-physiological explanations. Methodological explanations include (I) a higher concentration of clay in bulk soil compared to the rhizosphere soil, which could lead to a decreased DNA yield during DNA extraction (Novinscak and Filion, 2011). However, this should as well affect the rhizosphere of other crops grown in the same soil; (II) sugar beet root exudates enhance the growth of bacteria that are beneath the detection threshold in bulk soil or seeds. Ecological explanations for this phenomenon may include (III) soil bacteria and seed endophytes belonging to different genotypes both colonizing the rhizosphere, leading to an overall increased genetic diversity in the rhizosphere; (IV) a phylogenetic legacy. Sea beet (*Beta maritima*) as the progenitor of all beets is adapted to grow in sea drift lines, where abiotic selective forces like salinity, temperature, and UV exposure are more critical for plant survival than biotic stressors. Therefore, root exudates of beet seedlings may hardly contain compounds repellent to soil microbes and enrich microbes rather unspecifically. Given a temporal change in chemical root exudate composition, selective assembly of soil microbes may appear later in the life cycle once the seedling is established in its habitat; (V) the abundance of microbial responders toward sugar beet root exudates differ depending on the soil community. Since soils used in different studies differ in both biotic and abiotic properties, some soil microbiomes may be simply more responsive upon compounds exuded into the rhizosphere; (VI) It is a side effect of simple sugar content in root exudates, supporting trophic interactions between microbes. Sugar beet rhizospheres display a high abundance of polyhydroxyalkanoate (PHA) producing bacteria like *Burkholderia, Ensifer, Erwinia, Lysobacter, Pantoea, Pseudomonas*, and *Variovorax* (Gasser et al., 2009). This indicates an adaption of the bacterial community to excess carbon sources in combination with limiting other nutrient sources in the sugar beet rhizosphere (Lee, 1996). However, at the current stage, further meta-analyses or standardized longitudinal studies are needed to either support or falsify these hypotheses.

Appropriate nitrogen fertilization management in sugar beet is crucial for optimizing net sucrose yield. Available nitrogen is usually the plant growth-limiting nutrient. Although root biomass is positively correlated with N fertilization, sugar concentration in roots negatively correlates with N fertilization, resulting in an optimum curve regarding sucrose yield dependent on N application rate (Milford, 2006; Hergert, 2010). Consequently, fertilization regimes usually aim to let sugar beets become N-deficient several weeks before harvest to increase processing efficacy (Stevanato et al., 2016). Fertilization affects bacterial soil and rhizosphere microbiome community composition, including lowering the abundance of bacterial and archaeal nitrogen fixation-associated gene transcripts. Reduced nitrogen cycle-related gene abundance in sugar beet was observed when using chemical fertilization, alone or in combination with manuring (Cardinale et al., 2020). An indication for reduced nitrogen fixation with a concurrent increase of nitrification and aerobic ammonia oxidation in sugar beet rhizospheres was also found in a long-term crop rotation field (Du et al., 2022a). Archaeal nitrification in the rhizosphere further positively correlates with increasing soil depth (Stevanato et al., 2016).

Microbial rhizosphere communities shift during the maturation of the host plant. Sugar beet fibrous roots can reach up to 3 m depth (Stevanato et al., 2010), with the highest root length density at a depth of around 1 m (Stevanato et al., 2010, 2016). With the increasing length of the taproot, a vertical gradient of microbial diversity, increasing with depth, establishes alongside the fine roots under field conditions (Stevanato et al., 2016). *Proteobacteria* are enriched compared to bulk soil in pre-harvest taproot rhizospheres, *Acidobacteria, Actinobacteria, Bacteroidetes*, and *Gemmatimonadetes* were mentioned as codominant bacterial phyla (Houlden et al., 2008; Kusstatscher et al., 2019a; Cardinale et al., 2020; Huang et al., 2020; Du et al., 2022a). The fungal rhizosphere community of pre-harvest taproots is dominated by *Guehomyces, Humicola, Mortierella, Plectosphaerella*, and *Vishniacozyma* (Kusstatscher et al., 2019b; Du et al., 2022b), but is most likely less conserved than bacterial communities (Houlden et al., 2008).

Bacterial rhizosphere communities in sugar beet are dynamic across ontogenesis compared to other crops. Still, an increasingly crop-specific rhizosphere community establish over time (Houlden et al., 2008). As the physical, chemical, and biological activity of a plant itself shapes its surroundings even past its lifespan, continuous cropping of sugar beet severely affects soil traits. Continuous cropping usually leads to detrimental effects on yield and soil properties due to an imbalance in soil nutrients, autotoxicity of root exudates, and shifts in microbial community composition (Huang et al., 2020; Pervaiz et al., 2020; Cui et al., 2022). Effects in sugar beet include potassium depletion in soil (Samadi, 2012), rapid crop yield loss mainly due to plant disease intensification (Stevanato et al., 2019), and accumulation of potentially autotoxic root exudates (Huang et al., 2021). In fields where sugar beet was continuously grown for 0 (rotation cropping), 1, 5, and 30 years, bacterial and fungal alpha diversity was already lower after 1 year of sugar beet cultivation compared to planting in virgin soil but increased in year 30. This diversity increase however was attributable to potentially phytopathogenic species (*Acidobacteria, Alternaria, Fusarium, Cladosporium*) while beneficial genera (*Bacillus, Actinobacteria, Pseudomonas*) declined. The genera *Sphingomonas, Haliangium, Gaiella*, and *Lysobacter* were positively correlated, while *Lysinibacillus* and *Sphingobacterium* negatively correlated with sugar beet cropping time (Huang et al., 2020; Du et al., 2022a,b). It appears that long-time rotation with other crops does not fully restore the negative effects on sugar beet biomass, health parameters, and yield as well as on the community shift in bacterial (Cui et al., 2022; Du et al., 2022a) and fungal (Cui et al., 2022; Du et al., 2022b) rhizosphere microbiome compared to sugar beets grown in virgin soil. Strategies to re-establish the microbiome-related beneficial effects on plant growth found in virgin soil, while increasing the sustainability of the crop is a challenge of future sugar beet research.

#### 2.2.1. Root exudates fine-tune microbial communities in sugar beet rhizosphere

Root exudates are the main mechanism plants use for altering the microbial communities in their proximity while surrounding soil is the main source of diversity (Bais et al., 2006; Pantigoso et al., 2022). Furthermore, plant root exudation is used for recruiting pathogen antagonists from the environment (Cook et al., 1995; Mendes et al., 2011; Carrión et al., 2019). This assembly however may be specific to host traits and phylogeny. Transcriptomic profiling of *Ps. aeruginosa*-exposed root exudates of different sugar beet cultivars showed even cultivar-dependent responses (Mark et al., 2005). Additionally, the colonization patterns of sugar beet rhizosphere are strain-specific (Zachow et al., 2010); chemical root exudate composition shows spatially distinct patterns and may change when the root matures (Badri and Vivanco, 2009; Sasse et al., 2018) or when the root is exposed to nutrient deficiency (Khorassani et al., 2011). Over 200 compounds were measured in sugar beet root exudates, with salicylic acid and especially citramalic acid playing important roles in phosphor solubilization from the soil. Citramalic acid is rarely observed in plants and may even be produced by sugar beet-associated microbes (Khorassani et al., 2011). Root exudate chemical composition in sugar beet further appears to prevent close associations with arbuscular mycorrhizal fungi (Steinkellner et al., 2007). The root zone directly behind the root tip is considered the most active section regarding root exudate secretion (Sasse et al., 2018). A remarkably high proportion of *Variovorax paradoxus* (45%) was found in the cultivable fraction of lower root sections, but not in other endosphere compartments (1–4% in peel and inner core) (Lilley et al., 1996). The community in lower root sections was distinctly different from the soil, rhizosphere, peel, and inner core. *Variovorax* increases sugar beet biomass and was found to preferentially colonize root hair and the rhizodermis (Natsagdorj et al., 2019). Lübeck et al. (2000) tracked a fluorescein-labeled *Pseudomonas fluorescens* strain over 20 days during the germination of sugar beet seedlings. Strain density was 10-fold decreased at the root base within 1–2 days, while it remained stable and active at lower root parts and the root tip, again indicating an important role of root exudates in the rhizosphere and subsequently root endosphere colonization.

To summarize: the sugar beet rhizosphere microbiome is dominated by *Proteobacteria* and a dynamic habitat that is heavily influenced by the substrate. Microbes that should act as biofertilizers, biostimulants, or biocontrol agents (BCA) have to establish themselves in the rhizosphere. Thus, they should be copiotrophic and competitive to establish in this nutrient-rich and contested microhabitat. However, with a better understanding of the ecology of keystone species, including trophic interactions and other synergisms with other present microbiota, this statement can be challenged.

### 2.3. Gradients inside of the sugar beet taproot determine endophytic communities

The taproot of sugar beet is the actual agricultural target organ, where the rhizosphere community dynamics have the biggest impact on further plant health. Historically, three phases of vegetative development of sugar beet are distinguished: shoot growth, tuberization (storage root growth), and “ripening” (accumulation of sucrose in the tuber) (Milford, 2006). Only recently this classification could be proved by a detailed investigation of key enzyme profiles for carbohydrate metabolism and phytohormones patterns (Jammer et al., 2020). Apart from dynamics over time, the taproot itself has several internal spatially related gradients that could partially explain the responses of microbial communities that were observed so far.

#### 2.3.1. Root endophytes follow spatial and sucrose gradients

The sugar beet root simultaneously produces 6–7 concentric secondary cambia within the pericycle at an early seedling stage (Milford, 2006; Jammer et al., 2020). Sucrose is transported from leaves *via* vascular bundles to roots and then passively diffuses *via* the apoplast to the target root cell, which actively enriches sucrose in its vacuole (Wyse, 1979; Saftner et al., 1983). Therefore, sucrose concentration in the apoplast decreases with increasing distance to active secondary cambia, resulting in an increasing sucrose gradient from the outer to the inner core of taproots (Hoffmann and Kenter, 2018). This may explain why the cultivable fractions of endophytes are less diverse in the root core compared to the outer core, with a higher proportion of copiotrophs (Lilley et al., 1996; Okazaki et al., 2014). Okazaki et al. (2014) found *Pseudoxanthomonas, Polaromonas*, and *Devosia* in the outer core, and *Devosia, Bosea*, and *Lysobacter* to be dominant in the inner core. By contrast, another study (Lilley et al., 1996) found *Bacillus* and *Flavobacterium* to dominate the outer core, while *Pseudomonas* and *Bacillus* dominated the inner core. In the latter study, the number of cultivable endophytes of peel was even higher (1.09*10^8^ g^−1^) than in rhizosphere (5.27*10^7^ g^−1^), an effect that is not universal to root crops (Kõiv et al., 2019). However, a proximal-distal community shift was also observed using 16S rRNA amplicon sequencing of other root vegetables (Kõiv et al., 2019). Interestingly, the bacterial endosphere communities differ between taproots and secondary roots, with some taxa (e.g., *Niastella* spp.) being only found in secondary roots (Okazaki et al., 2021). In addition to a proximal-distal sucrose gradient, there is also a vertical gradient in the taproot: sucrose concentration is highest in the lower root part (16–20%) and decreases progressively from hypocotyl (ca. 15%) to lower (13%) and upper crown (7–9%). Decreasing sucrose concentration is accompanied by increased K, Na, and alpha-amino N (Milford, 2006). Interestingly, there is also a corresponding gradient of cultivable endophytes: the number of bacterial and fungal endophytes decrease from bottom to top (Jacobs et al., 1985; Shi et al., 2009a). That means the number of cultivable endophytes inside of taproots is correlated with apoplastic sucrose concentration as well as negatively correlated with spatial distance from soil (Figure 1B). However, since >90% of sugar beet-associated microbes appear to be non-cultivable (Lilley et al., 1996), these statements still need to be verified using cultivation-independent methods.

#### 2.3.2. Root endophytes shift during host ontogenesis

At harvest, the taproot consists of ca. 77% water, 17–18% sucrose, 4–5% marc (=insoluble compounds), and 1–2% water-soluble but non-sucrose compounds. Both dry matter and sugar content change during plant development, especially in the first 100 days post-sowing (Hoffmann et al., 2005). Harvest date, genotype, fertilizer, plant density, soil type, and seasonal weather can cause the proportion of dry matter and sucrose to vary (Milford, 2006). Secondary roots may act as a possible entrance for root endophytes (Jacobs et al., 1985; Shi et al., 2009b), but secondary roots are not desired by farmers.

Comparable to rhizosphere communities (Houlden et al., 2008), endophytic communities respond to seasonal variations. The number of cultivable taproot endophytes has an optimum in early fall (Shi et al., 2009a). Furthermore, the inner core of taproots significantly changes toward a lower diversity and lower evenness during taproot maturation due to an increasing abundance of *Bacillus* (Lilley et al., 1996). Shi et al. investigated sugar beet endophytes in four different life stages using amplicon sequencing. Bacterial (Shi et al., 2014), fungal (Shi et al., 2016), and archaeal (Shi et al., 2015) microbiomes of two cultivars at two different locations differed mainly due to developmental stage, rather than to cultivar or location. Life stage-dependent differences in bacteria were attributable to *Deltaproteobacteria, Nitrospirae, Acidobacteria, Gemmatimonadetes, Alphaproteobacteria*, and *Sphingobacteria* (Shi et al., 2014). By investigating bacterial communities only in the secondary roots of sugar beet across a growing season, a seasonal shift between life stages (Ikeda et al., 2023). They found some taxa that decrease in abundance after the seedling stage (e.g., *Janthinobacterium, Streptomyces*), some taxa to have an optimum during tuber growth (e.g., *Rhizobiaceae, Chitinophagaceae, Pseudomonas*), while some taxa increase across the whole plant ontogenesis until reaching an optimum during sucrose accumulation stage (e.g., *Steroidobacter*). Bacterial alpha diversity in secondary root endophytes increased until the late tuber growth phase and subsequently remained relatively stable (Ikeda et al., 2023). Longitudinal changes in endophytic or rhizosphere microbiomes may be linked to pathogen tolerance. Susceptibility toward plant pathogens decreases with the increasing age of the plant, irrespective if the cultivar itself is regarded as tolerant or susceptible to the pathogen (Liu et al., 2019). Current results indicate domain-specific diversity optima in different plant life stages: while bacterial diversity was highest during tuber growth (Shi et al., 2014; Ikeda et al., 2023), fungal diversity was highest during rosette formation (Shi et al., 2016), and archaeal diversity (dominant: *Methanococci, Crenarchaeota*, and *Thermoplasmata*) was highest during sucrose accumulation (Shi et al., 2015). However, the results of the mentioned studies are based on comparable small numbers of biological replicates and were not compared to the natural microbiome dynamics in bulk soil. Therefore, it is yet unclear if the observed microbiome shifts are due to a change in plant physiology (e.g., root exudation) or the natural seasonal dynamics in the corresponding bulk soil microbiome. During sugar beet harvest time, studies reported a high abundance and diversity of fungi, which significantly decreased in decaying sugar beets (Kusstatscher et al., 2019a,b). Fungal taxa such as *Candida* and *Penicillium* together with the gram-positive bacterium *Lactobacillus* were the main storage rot disease indicators in the microbiome of decaying sugar beets (see also Section 3.2).

#### 2.3.3. Nitrogen fertilization affects root endophytes

Sugar beet growing requires a comparably high amount of fertilizer, mainly nitrogen. Choosing an adequate amount of N fertilizer is crucial since too high concentrations of alpha-amino N in storage roots decrease the extractability of sucrose. Furthermore, nitrogen fertilizing increases leaf area (=photosynthetic potential) and size of root cells, but not the overall sugar content in the cell. Thus, bigger roots have a lower relative sugar concentration (Milford and Watson, 1971; Wyse, 1979; Hergert, 2010; Stevanato et al., 2019). In addition to the sucrose gradient in the apoplast depending on the distance to cambial structures, there is a reverse pattern regarding nitrogenous compounds and potassium ions, hypothesized to be important for sucrose transport to parenchymal cells (Milford, 2006). Similar to rhizosphere communities (Cardinale et al., 2020; see section 2.2), fertilizing affects the root endosphere bacterial community: in a bacterial metagenome study with chemical ammonium sulfate fertilization (Tsurumaru et al., 2015), the authors found several genes coding for PGP effects, but were not able to amplify known N_2_-fixating genes. In a follow-up study, a positive correlation between *Firmicutes* and nitrogen fertilization was observed, while *Sphingomonadaceae* was negatively correlated with nitrogen fertilization in the taproot endosphere (Okazaki et al., 2021). However, the endophyte community in the mentioned studies was dominated by *Rhizobiales*, mainly *Bradyrhizobium*, and *Mesorhizobium* (Tsurumaru et al., 2015; Okazaki et al., 2021). Given the importance of nitrogen sources for sugar beet growing, the importance of microbes in the soil nitrogen cycle, and the importance of root-internal alpha-amino N for post-harvest sucrose extraction, linking the right strategy for sugar beet nitrogen fertilization with the effects on and in different soil microbiomes needs further evaluation.

To summarize, the temporal and spatial dynamics during sugar beet development as well as the consequent changes in microbial communities highlight the importance of good sampling strategies for microbiome research. An elaborate and precise description of the considered plant organ or position on the beet, sampling procedure, plant developmental stage, available raw data, and metadata is crucial for the comparability of future results. Studies of different origins often report contradicting results, maybe owing to non-standardized sampling procedures, and have to be further evaluated.

### 2.4. Microbial assembly and dynamics in the sugar beet phyllosphere

The epiphyllosphere is generally thought to be a nutrient-scarce environment (Lindow and Brandl, 2003; Koskella, 2020), while the leaf interior is the main tissue of photosynthetic activity. Thus, epi- and endophyllospheres comprise very different conditions regarding UV exposure, temperature, nutrient availability, etc. Distinguishing between epi- and endophyllosphere is difficult because stomata and substomatal chambers directly connect the leaf interior and leaf exterior. Fluctuations in phyllosphere physicochemical conditions are thought to favor bacteria with versatile metabolic capabilities, which are often non-cultivable in the lab. However, pigmented microbes usually dominate leaf microbiomes (Lindow and Brandl, 2003; Bashir et al., 2022; Gouka et al., 2022).

Sugar beet phyllosphere microbiomes are subject to permanent fluctuations due to canopy dynamics, including leaf formation, senescence, and available habitat size in the apoplast due to changes in plant cell size and leaf size (Milford, 2006). In general, sugar beet plants produce new leaves as long as they remain in a vegetative state, and leaves usually wilt in the order they are developed. Leaves produced later in ontogenesis harbor higher bacterial endophyte diversity than earlier leaves, and the abundance of antagonists toward fungal phytopathogens is highest in a 90% leaf cover stage (Zachow et al., 2008).

*Pseudomonas* is the most frequently reported genus in sugar beet leaves. Using cultivation-dependent methods, other bacterial genera included *Arthrobacter, Clavibacter, Enterobacter, Erwinia*, and *Klebsiella* (Thompson et al., 1993, 1995b). Culture-independent analyses of sugar beet phyllosphere revealed the phyllosphere to contain a strikingly similar taxonomic composition compared to seed microbiomes. *Pseudomonas* is dominant (15–48% relative abundance), while *Sphingomonas, Pantoea, Methylobacterium*, and *Massilia* appear as codominant taxa (Wolfgang et al., 2020; Bertoldo et al., 2021; Della Lucia et al., 2021; Okazaki et al., 2021). This pattern is similar in both hydroponically grown and field-grown seedlings (Della Lucia et al., 2021). This points toward strong selective pressure in phyllosphere tissue for vertically transmittable endophytes even before seed formation, resulting in relatively conserved bacterial communities. With currently available data, it appears that phyllosphere bacterial diversity shifted and increased during domestication, while rhizosphere diversity decreased (see Section 2.6); sea beet phyllospheres are strongly dominated by *Sphingomonas* and *Methylobacterium*, together accounting for ca. 84% relative abundance (Broccanello et al., 2022). This however could as well refer to fertilization regimes, since bacterial phyllosphere microbiomes in sugar beet respond stronger upon NPK fertilization than in bacterial endosphere communities (Okazaki et al., 2021). Bacterial phyllosphere communities differ between petioles and laminae: *Phyllobacterium, Methylobacterium*, and *Sphingomonas* are higher-abundant, while *Pseudomonas* and *Enterobacteriaceae* are lower-abundant in petioles (Okazaki et al., 2021). Phyllosphere abundance of *Methylobacterium* and *Mucilaginibacter* was further linked to tolerance toward leaf pathogen *Cercospora beticola* in both sea beets and sugar beets (Broccanello et al., 2022). In contrast to bacteria, fungi isolated from sugar beet leaves usually belong to opportunistic plant pathogens (*Alternaria, Aspergillus, Cladosporium, Acremonium, Fusarium, Penicillium, Phoma, Plectosphaerella, Pleospora, Pythium*) but also saprobionts (*Cryptococcus, Epicoccum, Sporobolomyces, Stemphylium*) (Thompson et al., 1993; Larran et al., 2000; Shi et al., 2009a; Pusenkova et al., 2016). The number of cultivable filamentous fungi and yeasts increase in senescing leaves (Thompson et al., 1993), especially *Alternaria alternata* and *Pleospora herbarum* gradually increase during senescence (Larran et al., 2000). Senescent leaves and leaf debris will be degraded and incorporated into the soil together with their respective microbes, which is especially important for phytopathogen management.

### 2.5. Sugar beet anthosphere is a yet unexploited microhabitat

The formation of sugar beet flowers and seeds starts with bolting. Bolting usually requires vernalization but can also be induced spontaneously under cold weather conditions in the first year of growing (Owen et al., 1940; Milford, 2006). Bolting detracts sucrose from taproots, thus lowering yield. Sugar beet flowers are inconspicuous in appearance, which is typical for anemophilic plants. In the wild type, flowers merge into clusters, producing multigerm seedballs, which are fused corky fruits with up to five seeds (Lange et al., 1999; Panella and Lewellen, 2007). Seedballs are hypothesized to be an adaptation to hydrochory (Francis, 2006). Modern sugar beet cultivars display monogermity (seedball with one single embryo), which is a recessive maternal trait in beets. Consequently, modern sugar beet cultivars are usually 2- or 3-way hybrids (Panella and Lewellen, 2007). Hybridization and breeding history influence plant-microbiome interactions (see Section 2.6). Counterintuitively, offspring microbiomes can resemble rather the microbiomes of the pollen-providing parent than the seed-bearing mother plant, which was recently found in specific oilseed rape (*Brassica napus* L.) cultivars (Wassermann et al., 2022), but the exact implications of parent sex and respective microbiomes on offspring microbiomes has yet to be elucidated in sugar beet. Therefore, anthosphere microbiome dynamics may have yet unknown potential for microbiome-based breeding approaches.

Flowering lasts 1 month or longer, and sugar beet pollen can be transported across large distances by wind (Darmency et al., 2009). Consequently, both stamen and pollen are exposed considerably long to environmental factors including horizontally transmitted microbiota. Sugar beet pollination does not require insects. Still, flowers produce nutrient-rich pollen and odorous nectar, attracting several insect taxa that transport pollen (Free et al., 1975) or may transmit microbes to flowers and subsequently seeds (Hardoim et al., 2015; Berg and Raaijmakers, 2018; Nelson, 2018). However, such microbes will not form close relationships with the host plant, unless they can adapt to the selective force of the spermosphere (see Section 2.1). Nevertheless, anthosphere microbiome modulation, e.g., by introducing beneficial seed endophytes during flowering before seed maturation, may as well have the potential to promote plant health of the offspring generation.

### 2.6. The impact of domestication on the sugar beet microbiome

Plant domestication changes the morphology, physiology, and genetic potential of a given species, but also unintentionally shapes associated microbiomes via host-mediated, multi-generational microbiome selection (Johnston-Monje and Raizada, 2011, 2013; Orozco-Mosqueda et al., 2018; Pérez-Jaramillo et al., 2018). Sea beet, as the wild ancestor of sugar beet, is frequently used for comparative studies and backcrossing approaches to increase tolerance of modern cultivars toward biotic (Whitney, 1989), and abiotic stresses (Monteiro et al., 2013; Stevanato et al., 2019). Sea beet however carries traits undesired by breeders, namely annual life cycle, red pigments in root tissues, fangy or sprangled roots, multigerm flowers, elongated crowns, multiple crowns, low sucrose concentration, and low sucrose extractability (Panella and Lewellen, 2007). The Silesian beet was the first sugar beet line and was bred out of fodder beet and chard in the late 18th century (Fischer, 1989). Soon breeding started to diverge, resulting in cultivars of the Z-type (high sugar content), the E-type (optimized for biomass), and the N-type (intermediate type) (Francis, 2006). Ongoing breeding activities led to an increase in sugar concentration in sugar beet tap roots from 12 to ca. 18% (Milford, 2006; Dohm et al., 2014). Sugar yield continuously increased, attributable to higher average temperatures in spring (Jaggard et al., 2007), improved management practices (Hoffmann and Kenter, 2018), and most importantly breeding progress (Hoffmann and Loel, 2015).

Crop breeding in general mainly focuses on genomic traits, leading to higher yields, but also genetic homogeneity and erosion of genetic diversity (Gopal and Gupta, 2016). Due to the historically recent development of sugar beet, some authors regard the genetic background of sugar beet as relatively narrow compared to several other crops (Bosemark, 1979; Fischer, 1989; Stevanato et al., 2019; Galewski et al., 2022). Still, intentional as well as unintentional admixture and introgression events in the course of domestication and breeding re-introduced considerable genetic variation in beet crops (Galewski and McGrath, 2020). Nevertheless, the process of sugar beet domestication is correlated with a community shift in bacteria: bacterial rhizosphere communities of sea beet have a significantly higher alpha diversity and evenness than modern sugar beet cultivars when grown in the same substrate (Zachow et al., 2014). Such domestication-related effects were observed in other plant species like wheat (Germida and Siciliano, 2001), and beans (Pérez-Jaramillo et al., 2017) as well. Decreasing *Bacteroidetes* abundance in the rhizosphere was observed in several crops, including sugar beet, as a consequence of domestication (Pérez-Jaramillo et al., 2018). Differences between root community structure and its corresponding soil are bigger in modern sugar beets compared to sea beets; modern sugar beet roots have lesser but stronger responders to root exudates already in the seedling state (namely *Novosphingobium, Pseudomonas, Sphingomonas*). These differences in rhizosphere bacteria may have implications for soil-borne disease pathogenesis: bacterial isolates extracted from sea beet roots contained on average fewer antagonists of fungal phytopathogens but displayed higher tolerance toward salt stress when compared to isolates from sugar beet (Zachow et al., 2014). Since bacterial alpha diversity is often positively correlated with pathogen tolerance (Berg et al., 2017; Du et al., 2022a), the microbiome itself may also contribute to the resilience of the sea beet. In addition, the coastline as natural habitat itself may contain low pathogen pressure compared to osmotic pressure, which again may emphasize the reciprocal adaption of the host and its microbiome within its natural habitat. For sugar beet farming, however, high microbial rhizosphere alpha diversity may not always increase yield under different field conditions or in different cultivars. Some microbiome-mediated services to plants, e.g., stress resilience, nutrient acquisition, or phytopathogen antagonism, may not increase plant performance if conditions are already optimal or the corresponding stress is absent. The advantage of high microbial alpha diversity in the rhizosphere is usually more apparent under suboptimal growing conditions. To re-introduce resilience to the sugar beet crop using bacterial diversity, the lower alpha diversity in modern cultivars indicate actually a potential for intentionally introduced beneficial microorganisms (e.g., via seed priming) to establish in the rhizosphere, since some micro-niches in roots may not be occupied. In general, implementing functional hologenomic in breeding programs holds the potential to further increase desired plant traits and field performances of a crop (Nogales et al., 2016).

## 3. Part II: Microbiome management and pathogen control in sugar beet

### 3.1. Modulating sugar beet-associated microbiomes in the field

Using microbes to improve plant performance and health is a promising approach for the agriculture of the 21st century, not only in sugar beet. Several management options are available to modulate microbial communities in the field, including physical, chemical, biological, and logistical practices. In general, biological options include single strains (Section 3.1.1) or consortia (Section 3.1.2) with bioactive properties, the use of microbiota-active metabolites, application of microbiome transplants, or changing the environmental setting to indirectly shift the structure and functions of microbiomes (Berg and Smalla, 2009; Orozco-Mosqueda et al., 2018; Berg et al., 2021). Seed endophytes are of special interest for agricultural applications and targeted microbiome modulations (Berg and Raaijmakers, 2018; Shahzad et al., 2018) because of their vertical transmission, but environmental microbiomes known for high resilience toward biotic and abiotic stress can be exploited as well (Zachow et al., 2013). Methods to apply microorganisms include drenching, soil amendments, spraying, seed or seedling inoculation, tissue atomization, and direct injection. The upcoming sections discuss several examples of microbiome-based control, ranging from the application of single BCAs (Section 3.1.1) and consortia (Section 3.1.2) to the potential of soil amendments (Section 3.1.3) and disease-suppressive soils (Section 3.1.4) to positively influence sugar beet vigor.

#### 3.1.1. Sugar beet biocontrol and plant growth promotion are available

Applying microbes to sugar beet usually aims at suppressing pathogens in the field to increase yield, and antagonism toward pathogens is often correlated with plant growth promotion (PGP, Table 1). Organic farming of sugar beet is challenging at market prices, amongst others due to insufficient availability, and missing or scattered scientific knowledge on environmental-friendly alternatives for pathogen suppression (Stevanato et al., 2019). Important pathogens of sugar beet include mainly fungal pathogens (Table 1), especially different anastomosis groups of *Rhizoctonia solani* J.G.KÜHN [teleomorph: *Thanatephorus cucumeris* (A.B.Frank) Donk], *Cercospora beticola* SACC., and the oomycete *Globisporangium ultimum* (Trow) Uzuhashi, Tojo and Kakish. (syn.: *Pythium ultimum*). Diseases caused by these pathogens include crown rot, damping-off, and early and late root rot. As an example, late root rot, a disease caused by *R. solani* AG2-2IIIB, can lead locally to yield losses >50% on the field or during storage at sugar refineries. It is estimated to affect 24% of the acreage in the USA, and 5–10% in Europe (Büttner et al., 2004; Jacobsen, 2006; Windels et al., 2009). First sugar beet resistance to *Rhizoctonia* root rot was discovered in breeding lines developed in Fort Collins, USA (Panella et al., 2015), but these cultivars often insufficiently carried other desired traits like bolting resistance, yield or processing quality (Büttner et al., 2004). Nowadays, *Rhizoctonia*-tolerant varieties have been improved significantly thanks to breeding progress and additionally provide protection against other plant diseases. Since *Rhizoctonia* tolerance is a polygenic trait (Galewski et al., 2022) and due to its quantitative inheritance however, yield potential could not be improved as fast as for single gene-mediated tolerances, e.g., resistance against beet necrotic yellow vein virus (BNYVV) causing rhizomania (Scholten and Lange, 2000), or beet cyst nematodes (*Heterodera schachtii* A.Schmidt) (Cai et al., 1997). In seeds, the genus *Kosakonia* seems to be correlated with *Rhizoctonia*-tolerant sugar beet cultivars (Wolfgang et al., 2020), but further investigations are needed to generalize this correlation. Anyway, using biocontrol and plant growth-promoting microbes has considerable potential, especially in susceptible cultivars providing high yields.

**Table 1:** Biocontrol of selected phytopathogens and plant growth promotion potential of different biocontrol agents (BCAs).

**Applied organism (strain):**	**Application**	**Cultivar**	**Pathogen/pest**	**Effects**	**Reference**
**Bacteria**					
**Bacillaceae**					
*Bacillus amyloliquefaciens* SS-38.4	Syringe injection with crude lipopeptide extract or cell suspensions (10^7^ CFU/ml)	cv. Marinela, cv. Serenada, cv. Jasmina, cv. Lara	*Pseudomonas syringae* pv. *aptata*	Inhibition of leaf tissue necrosis	Nikolić et al., 2019
*Bacillus amyloliquefaciens*	5 x soil drenching with 3.125*10^8^ cells/plant	cv. Barrosa	*Sclerotium rolfsii*	Increased shoot and root length, fresh weight, dry weight, 63% control efficacy	Farhaoui et al., 2022
*Bacillus amyloliquefaciens* S499	Watered with cyclic lipopeptides extract	cv. Cadyx	*Polymyxa betae*	Induced systemic resistance, reduced infection (qPCR)	Desoignies et al., 2013
*Bacillus amyloliquefaciens* SB14	Seed priming with 9*10^8^ CFU/mL in sterile carboxymethyl cellulose (CMC) 1% solution.	cv. Shirin	*Rhizoctonia solani*	Damping-off reduction	Karimi et al., 2016
*Bacillus amyloliquefaciens* SS-12.6	syringe injection with crude lipopeptide extract or cell suspensions (10^7^ CFU/ml)	cv. Marinela, cv. Serenada, cv. Jasmina, cv. Lara	*Pseudomonas syringae* pv. *aptata*	Inhibition of leaf tissue necrosis	Nikolić et al., 2019
*Bacillus brevis*	Drenching with bacterial suspension (25ml of 10^7^ CFU/ml)	cv. HH77	*Heterodera schachtii*	Reduces nematodes /g root	Neipp and Becker, 1999
*Bacillus halotolerans*	5 x soil drenching with 3.125*10^8^ cells/plant	cv. Barrosa	*Sclerotium rolfsii*	Increased shoot and root length, fresh weight, dry weight, 73% control	Farhaoui et al., 2022
*Bacillus megaterium*	Drenching with bacterial suspension (25ml of 10^7^ CFU/ml)	cv. HH77	*Heterodera schachtii*	Reduces nematodes /g root	Neipp and Becker, 1999
*Bacillus megaterium P8S105*	Seed priming (unknown concentration)	NA	*Globisporangium (= Pythium) ultimum, Aphanomyces cochlioides*	Improved emergence, increase healthy seedlings	Williams and Asher, 1996
*Bacillus mycoides* Bac J	Syringe infiltration of live cells in primary leaves	cv. C40, cv. USH11	*Cercospora beticola*	Induction of oxidative burst, disease reduction	Bargabus et al., 2003
*Bacillus mycoides* Bac J	Spray-dried formulation of dead or alive Bacillus mycoides BmJ suspended in water (10^7^ CFU/ml); leaf spray on oldest leaves with aerosol	cv. Holly Hybrid 88, cv. Seedex 920002, cv. Beta 1996, cv. VDH 66140, cv. KW2262, cv. Beta 2185	*Cercospora beticola*	Field and glasshouse experiments, induced systemic resistance, reduced leaf spot disease severity and symptoms	Bargabus et al., 2002
*Bacillus pumilus*	Drenching with bacterial suspension (25ml of 10^7^ CFU/ml)	cv. HH77	*Heterodera schachtii*	Reduces nematodes /g root	Neipp and Becker, 1999
*Bacillus pumilus* 203-6	Aerosol spray to penultimate true leaf, 10^8^ CFU/ml	cv. Holly Hybrid 88, cv. Seedex 920002	*Cercospora beticola*	Reduced *Cercospora* leaf spot symptoms	Bargabus et al., 2004
*Bacillus pumilus* 203-7	Aerosol spray to penultimate true leaf, 10^8^ CFU/ml	cv. Holly Hybrid 88, cv. Seedex 920002	*Cercospora beticola*	Reduced *Cercospora* leaf spot symptoms	Bargabus et al., 2004
*Bacillus pumilus* SB6	Seed priming with 9*10^8^ CFU/mL in sterile carboxymethyl cellulose (CMC) 1% solution.	cv. Shirin	*Rhizoctonia solani*	Damping-off reduction	Karimi et al., 2016
*Bacillus pumilus* SS-10.7	Syringe injection with crude lipopeptide extract or cell suspensions (10^7^ CFU/ml)	cv. Marinela, cv. Serenada, cv. Jasmina, cv. Lara	*Pseudomonas syringae* pv. *aptata*	Inhibition of leaf tissue necrosis	Nikolić et al., 2019
*Bacillus safensis*	5 x soil drenching with 3.125*10^8^ cells/plant	cv. Barrosa	*Sclerotium rolfsii*	Increased shoot and root length, fresh weight, dry weight, 17% control efficacy	Farhaoui et al., 2022
*Bacillus siamensis* AP2	Seed priming with 9*10^8^ CFU/mL in sterile carboxymethyl cellulose (CMC) 1% solution.	cv. Shirin	*Rhizoctonia solani*	Damping-off reduction	Karimi et al., 2016
*Bacillus siamensis* AP8	Seed priming with 9*10^8^ CFU/mL in sterile carboxymethyl cellulose (CMC) 1% solution.	cv. Shirin	*Rhizoctonia solani*	Damping-off reduction	Karimi et al., 2016
*Bacillus subtilis*	5 x soil drenching with 3.125*10^8^ cells/plant	cv. Barrosa	*Sclerotium rolfsii*	Increased shoot and root length, fresh weight, dry weight, 6-63% control efficacy	Farhaoui et al., 2022
*Bacillus* sp. (“*cytaseus*”) C-82	Seed priming (10^7^ cells/ml)	NA	*Fusarium oxysporum, Alternaria alternata*	Decreased damping-off, increased germination rate, shoot length, and root length	Smirnova and Sadanov, 2019
**Comamonadaceae**					
*Variovorax paradoxus*	Drenching with bacterial suspension (25ml of 10^7^ CFU/ml)	cv. HH7	*Heterodera schachtii*	Reduces nematodes /g root	Neipp and Becker, 1999
**Enterobacteriaceae**					
*Erwinia carotovora* pv. *betavasculorum* (avirulent)	Syringe infiltration of live cells in primary leaf	cv C40, cv. USH11	*Cercospora beticola*	Induction of oxidative burst, disease reduction	Bargabus et al., 2003
*Serratia plymuthica* 3Re4-18	Consortia seed priming	cv. Belladonna, cv. Beretta, cv. Isabella, cv. Laetitia, cv. Mattea	*Rhizoctonia solani*	Increased number of healthy beets in susceptible cultivars, decreased disease index	Wolfgang et al., 2020
**Flavobacteriaceae**					
*Flavobacterium (Cytophaga) johnsoniae* P1T139	Seed priming (unknown concentration)	NA	*Globisporangium (=Pythium) ultimum, Aphanomyces cochlioides*	Improved emergence, increase healthy seedlings	Williams and Asher, 1996
**Methylophilaceae**					
*Methylovorus mays* BKM B-2221	Seedling dipping of cell suspension, leaf spray	cv. Yaltushkovskaya 34, cv. L'govskaya 52, cv. L'govskaya 187p47, cv. L'govskaya 16	*Erwinia carotovora*	Increased pathogen tolerance and root regeneration, plant growth promotion, increased growth speed and photosynthetic activity	Pigoleva et al., 2009
**Micrococcaceae**					
*Paenarthrobacter histidinolovorans* P2T9	Seed priming (unknown concentration)	NA	*Globisporangium (=Pythium) ultimum, Aphanomyces cochlioides*	Improved emergence, increase healthy seedlings	Williams and Asher, 1996
*Paenarthrobacter oxydans*	Drenching with bacterial suspension (25ml of 10^7^ CFU/ml)	cv. HH77	*Heterodera schachtii*	Reduces nematodes /g root	Neipp and Becker, 1999
*Arthrobacter ramosus*	Drenching with bacterial suspension (25ml of 10^7^ CFU/ml)	cv. HH77	*Heterodera schachtii*	Reduces nematodes /g root	Neipp and Becker, 1999
**Pasteuriaceae**					
*Pasteuria nishizawae*	Suspension, spore suspension, 4.1*10^6^/L soil, 5*10^8^/L soil	cv. Beretta, cv. Sanetta, cv. Pauletta	*Heterodera schachtii*	Egg masses decrease, induction of suppressiveness and yield increase over 3 years	Eberlein et al., 2020
**Pseudomonadaceae**					
*Pseudomonas alcaligenes*	Drenching with bacterial suspension (25ml of 10^7^ CFU/ml)	cv. HH77	*Heterodera schachtii*	Reduces nematodes /g root	Neipp and Becker, 1999
*Pseudomonas brassicacearum* L13-6-12	Consortia seed priming	cv. Belladonna, cv. Beretta, cv. Isabella, cv. Laetitia, cv. Mattea	*Rhizoctonia solani*	Increased number of healthy beets in susceptible cultivars, decreased disease index	Wolfgang et al., 2020
*Pseudomonas corrugata* R117	Seed priming (10^5^-10^6^ CFU/seed)	cv. Bianca	*Globisporangium (=Pythium) ultimum*	Damping-off reduction	Georgakopoulos et al., 2002
*Pseudomonas fluorescens* 54	50 μl suspension (10^8^ CFU/ml) directly pipetted on seed in soil	cv. Magnat	*Globisporangium (=Pythium) ultimum*	Increased healthy seedlings 14 days post-sowing,	Nielsen et al., 1998
*Pseudomonas fluorescens* B5	Seed priming (10^5^-10^6^ CFU/seed)	cv. Bianca	*Globisporangium (=Pythium) ultimum*	Damping-off reduction	Georgakopoulos et al., 2002
*Pseudomonas fluorescens* DR54	Seed dipping (suspension directly on seed, field experiment) and seed priming (4*10^9^, pot and microcosm experiments)	cv. Madison	*Rhizoctonia solani* AG4	Increases emergence 24 days post-sowing, health of seedlings, leads to reduction of radial extension of mycelium, hyphae start to branch, decreased biomass of pathogen	Thrane et al., 2000
*Pseudomonas fluorescens* DR54	Seed priming with 5*10^7^ CFUs/seed	cv. Madison	*Pythium* spp.	Reduce emergence when not challenged but increase when challenged, reduced root length when challenged,	Thrane et al., 2000
*Pseudomonas fluorescens* F113 lacZY	Alginate beads (comprising 10^6^) compared to free cells seed priming	cv. Golf	*Globisporangium (=Pythium) ultimum, Rhizoctonia solani*	Increased emergence	Russo et al., 2001
*Pseudomonas fluorescens* F113Rif					
*Pseudomonas fluorescens* ML5	Seed priming (10^7^-10^8^ CFU/seed) and talc	USH11	*Globisporangium (=Pythium) ultimum*	Reduced fungal colonization, reduced viable mycelium in pericarp, inhibition of mycelial growth and sporangial germination	Osburn, 1989
*Pseudomonas fluorescens* P22P101	Seed priming (unknown concentration)	NA	*Globisporangium (=Pythium) ultimum, Aphanomyces cochlioides*	Improved emergence, increase healthy seedlings	Williams and Asher, 1996
*Pseudomonas fluorescens* X	Seed priming (10^5^-10^6^ CFU/seed)	cv. Bianca	*Globisporangium (=Pythium) ultimum*	Damping-off reduction	Georgakopoulos et al., 2002
*Pseudomonas poae* RE*1-1-14	Consortia seed priming	cv. Belladonna, cv. Beretta, cv. Isabella, cv. Laetitia, cv. Mattea	*Rhizoctonia solani*	Increased number of healthy beets in susceptible cultivars, decreased disease index	Wolfgang et al., 2020
*Pseudomonas putida* R20	Seed priming (10^7^-10^8^ CFU/seed) and talc	USH12	*Globisporangium (=Pythium) ultimum*	Reduced fungal colonization, reduced viable mycelium in pericarp, inhibition of mycelial growth and sporangial germination	Osburn, 1989
*Pseudomonas syringae* P22P104	Seed priming (unknown concentration)	NA	*Globisporangium (=Pythium) ultimum, Aphanomyces cochlioides*	Improved emergence, increase healthy seedlings	Williams and Asher, 1996
**Streptomycetaceae**					
*Kitasatospora aureofaciens*	Seed coating, soil pre-inoculation, seed soaking	cv. Raspoly, cv. TOP, cv. Tribel	*Fusarium solani*	Partially increased emergence, decreased infection, partially increased root and shoot length	Moussa and Rizk, 2002
*Streptomyces* sp. B-11	Soil drenching of cell suspension	NA	*Sclerotium rolfsii*	*Sclerotium* inhibition, hyphal inhibition, increased fresh weight	Errakhi et al., 2009
Streptomyces sp. C	seed coating, 10^6^/ml	cv. Shirin	*Rhizoctonia AG-4*	84% damping-off disease reduction, increased root and shoot weight, sucrose yield	Sadeghi et al., 2009
Streptomyces sp. “C”	seed priming in 10^6^/ml	cv. Shirin	*Rhizoctonia AG-2, Fusarium solani, Phytophthora drechsleri*	*in vitro* and *in vivo* reduction of pathogen growth, decreased disease incidence	Karimi et al., 2012
*Streptomyces* sp. J-2	Soil drenching of cell suspension	NA	*Sclerotium rolfsii*	*Sclerotium* inhibition, hyphal inhibition, increased fresh weight	Errakhi et al., 2009
Streptomyces sp. S2	Seed coating, 10^6^/ml	cv. Shirin	*Rhizoctonia AG-4*	77% damping-off disease reduction, increased root and shoot weight, sucrose yield	Sadeghi et al., 2009
**Xanthomonadaceae**					
Lysobacter sp. SB-K88	Seed priming (10^8^/seed), pathogen added after 2 weeks	cv. Abendrot	*Aphanomyces cochlioides*	Increased % healthy seedlings	Islam et al., 2005
Lysobacter sp. SB-K88	Seed priming (10^8^/seed)	cv. Monoace S	*Pythium* spp.	Suppression of damping-off	Nakayama et al., 1999
*Lysobacter enzymogenes* C3	seed priming (5*10^8^) with 1% methylcellulose	NA	*Globisporangium (=Pythium) ultimum*	increased emergence in infested soil	Kobayashi et al., 2005
*Lysobacter enzymogenes* C3	The bacterial strains were coated onto sugar beet seed (10^7^ CFU/seed) as cell suspensions in 1% methylcellulose.	NA	*Globisporangium (=Pythium) ultimum*	Increased fraction of emerging and surviving seedlings	Palumbo et al., 2005
*Stenotrophomonas maltophilia* W81	Seed inoculation	cv.Accord	*Globisporangium (=Pythium) ultimum*	Increased percentage of *Pythium*-free seeds, increased plant emergence	Dunne et al., 2000
**Fungi (Ascomycota)**					
**Aspergillaceae**					
*Penicillium pinophilum*	Soil drenching 2*10^4^ spores/plant	cv. Crystal 101RR	*Rhizoctonia solani* AG2-2 IIIB	66% reduction of damping-off	Haque et al., 2021
**Clavicipitaceae**					
*Metacordyceps* (*Pochonia*) *chlamydosporia* Pc001	Soil amendment of agar plugs	cv. Beretta	*Heterodera schachtii*	Egg parasitizing fungi, increased root weight compared to infected beets, propagated cysts, infected eggs	Haj Nuaima et al., 2021
**Cucurbitariaceae**					
*Pyrenochaeta* sp. Py004	Soil amendment of agar plugs	cv. Beretta	*Heterodera schachtii*	Egg parasitizing fungi, increased root weight compared to infected beets, propagated cysts, infected eggs	Haj Nuaima et al., 2021
**Debaryomycetaceae**					
*Candida valida*	Soil drenching 2 weeks pre-infection	cv. Maribo Magna	*Rhizoctonia solani* AG2-2	Decreased post-emergence damping-off, decreased crown and root rot symptoms, increased root and shoot fresh weight	El-Tarabily, 2004
**Herpotrichiellaceae**					
*Exophiala* sp. Ex007	Soil amendment of agar plugs	cv. Beretta	*Heterodera schachtii*	Egg parasitizing fungi, increased root weight compared to infected beets, propagated cysts, infected eggs	Haj Nuaima et al., 2021
**Hypocreaceae**					
*Trichoderma atroviride* I-2	Seed priming overnight plus watering with 2ml conidia suspension (10^8^/ml), pot experiment	cv. Intera	*Polymyxa betae*, BNYVV	Reduction of *P. betae* cystosori in roots, inhibition of BNYVV replication	Jakubíková et al., 2006
*Trichoderma atroviride* Tr-3	Seed priming 2h	cv. Arosa	BNYVV	Reduced BNYVV antibody absorbance in infected roots (DAS-ELISA). Reduction of *Polymyxa betae* resting spores not significant	Yilmaz and Tunali, 2010
*Trichoderma harzianum* I-1	Seed priming overnight plus watering with 2ml conidia suspension (10^8^/ml), pot experiment	cv. Intera	*Polymyxa betae*, BNYVV	Reduction of *P. betae* cystosori in roots, inhibition of BNYVV replication	Jakubíková et al., 2006
*Trichoderma harzianum* I-3	Seed priming overnight plus watering with 2ml conidia suspension (10^8^/ml), pot experiment	cv. Intera	*Polymyxa betae*, BNYVV	Reduction of *P. betae* cystosori in roots, inhibition of BNYVV replication	Jakubíková et al., 2006
*Trichoderma harzianum* K10	Seed coating, 10^6^/ml	cv. Shirin	*Rhizoctonia solani* AG-4	66% damping-off disease reduction, increased root and shoot weight, sucrose yield	Sadeghi et al., 2009
*Trichoderma harzianum* Tr-7	Seed priming 2h	cv. Arosa	BNYVV	Reduced BNYVV antibody absorbance in infected roots (DAS-ELISA). Reduction of *Polymyxa betae* resting spores not significant	Yilmaz and Tunali, 2010
*Trichoderma harzianum* Tr-8	Seed priming 2h	cv. Arosa	BNYVV	Reduced BNYVV antibody absorbance in infected roots (DAS-ELISA). Reduction of *Polymyxa betae* resting spores not significant	Yilmaz and Tunali, 2010
*Trichoderma koningii* Tr-9	Seed priming 2h	cv. Arosa	BNYVV	Reduced BNYVV antibody absorbance in infected roots (DAS-ELISA). Reduction of *Polymyxa betae* resting spores not significant	Yilmaz and Tunali, 2010
*Trichoderma longibrachiatum* MHC 22	Seed priming overnight plus watering with 2ml conidia suspension (10^8^/ml), pot experiment	cv. Intera	*Polymyxa betae*, BNYVV	Reduction of *P. betae* cystosori in roots, inhibition of BNYVV replication	Jakubíková et al., 2006
*Trichoderma viride* Tr-4	Seed priming 2h	cv. Arosa	BNYVV	Reduced BNYVV antibody absorbance in infected roots (DAS-ELISA). Reduction of *Polymyxa betae* resting spores not significant	Yilmaz and Tunali, 2010
**Orbiliaceae**					
*Hyalorbilia* aff. *multiguttulata* DoUCR50	Suspension, spore suspension, 4.1*10^6^/L soil, 5*10^8^/L soil	cv. Beretta, cv. Sanetta, cv. Pauletta	*Heterodera schachtii*	Egg masses decrease, induction of suppressiveness and yield increase over 3 years	Eberlein et al., 2020
**Xylariaceae**					
*Muscodor albus* 620	0.5g/60cm^3^ infested soil of starch and corn oil-based formulation; ground barley formulation	Beta 8754	*Globisporangium (=Pythium) ultimum, Rhizoctonia solani, Aphanomyces cochlioides, Meloidogyne incognita*	Increased percentage of healthy seedlings 28 days post-planting,	Grimme et al., 2007
**Fungi (Basidiomycota)**					
**Corticiaceae**					
*Laetisaria arvalis*	Soil amendment (222kg/ha)	NA	*Rhizoctonia solani*	hyperparasite, decreases *Rhizoctonia* in the field	Allen et al., 1985
**Sporidiobolaceae**					
*Rhodotorula glutinis*	Soil drenching 2 weeks pre-infection	cv. Maribo Magna	*Rhizoctonia solani* AG2-2	Decreased post-emergence damping-off, decreased crown and root rot symptoms, increased root and shoot fresh weight	El-Tarabily, 2004
**Trichosporonaceae**					
*Trichosporon asahii*	Soil drenching 2 weeks pre-infection	cv. Maribo Magna	*Rhizoctonia solani* AG2-2	Decreased post-emergence damping-off, decreased crown and root rot symptoms, increased root and shoot fresh weight	El-Tarabily, 2004
**Oomycota**					
**Pythiaceae**					
*Pythium oligandrum*	Seedling root priming with cell wall proteins	cv. Megumi	*Globisporangium (=Pythium) ultimum*	Reduction of root necrosis (55% and 30.4%)	Takenaka et al., 2006
*Pythium oligandrum*	Oospore seed coating (12500/seed)	cv. USDA breeding line V137 H8	*Globisporangium (=Pythium) ultimum*	Improved seedling emergence, decreased preemergence damping-off	Martin, 1987
*Pythium oligandrum*	oospore seed coating	cv. Amethyst	*Globisporangium (=Pythium) ultimum, Aphanomyces cochlioides*	reduction of damping off	McQuilken et al., 1990

Microbes already adapted to an endophytic lifestyle or a specific host plant appear to be more effective in biocontrol and PGP. For instance, strains isolated from sugar beet rhizosphere were reported to outperform BCAs isolated from other plant hosts in *Rhizoctonia*-infested sugar beet (Karimi et al., 2016). On the other hand, sugar beet-adapted microbiota can also show positive effects in other crops (Natsagdorj et al., 2019). Endophytes were frequently successfully tested for sugar beet PGP, including strains of *Acinetobacter, Achromobacter, Bacillus, Burkholderia, Chryseobacterium*, and *Pseudomonas*. Positive effects include increased fresh weight, dry weight, number of leaves, absorption of K, N, Mg, and P, Vitamin B and C content, leave size, germination rate, plant height, shortened germination time, and lower tissue water content (Çakmakçi et al., 2001; Shi et al., 2009b, 2011, 2017; Gasser et al., 2011; Piernik et al., 2017). Nevertheless, it may be reasonable to isolate microbes from non-target hosts or environments adapted to given stressors. Zachow et al. (2013) used sugar beet plants to enrich bacterial taxa from substrate amended with either a pH-tolerant moss species, cold-adapted primroses, or drought-adapted lichens for targeted extraction of strains both mediating tolerance toward abiotic stress and compatible with the host plant. Such crop-adapted microbiome transplants hold the big potential to isolate strains that increase crop resilience toward the numerous biotic and abiotic challenges agriculture will face during the Anthropocene (Berg and Cernava, 2022).

Most biocontrol approaches to control phytopathogens use members of either *Bacillus* or *Pseudomonas* solely applied as seed priming (Table 1). Both genera carry several beneficial traits regarding agricultural applications, e.g., the ability to produce endospores. Furthermore, the importance of *Pseudomonas* in disease-suppressive soils (Mendes et al., 2011), as well as in sugar beet-associated bacterial communities, was frequently reported (Thompson et al., 1993; Zachow et al., 2008; Wolfgang et al., 2020; Okazaki et al., 2021), especially fluorescent pseudomonads (Thompson et al., 1993, 1995a,b; Rainey et al., 1994; Lilley et al., 1997; Ellis et al., 1999). *Pseudomonas* strains produce interesting secondary metabolites with antifungal properties, e.g., lipopeptides like amphisin, poaeamide, viscosin, and viscosinamide (Andersen et al., 2003; Zachow et al., 2015). There is evidence that some *Pseudomonas* adapt lipopeptide and endochitinase production when exposed to different sugar concentrations (Nielsen et al., 1998), which is advantageous regarding the variable sugar concentration in sugar beet.

Biocontrol of beet pathogens was even achieved with subcellular components of BCAs. For instance, reduced root necrosis caused by *G. ultimum* was observed when seedlings were primed with only cell wall proteins of *Pythium oligandrum* DRECHSLER, most likely triggering host defense response (Takenaka et al., 2003). To summarize, a high number of potential BCAs in sugar beet is readily available and scientifically investigated, but has yet to be established in current agricultural practices.

#### 3.1.2. Consortia vs. single strains in seed priming: pros and cons

Combining and applying different BCA strains as consortiums can have additive or synergistic effects on plant health (Carrión et al., 2019). *Verticillium* wilt symptoms were reduced using *Verticillium tricorpus* and non-pathogenic *Fusarium oxysporum* as soil amendments under greenhouse conditions (Lopisso et al., 2017). Another study reported a yield increase, an increase in the numbers of healthy beets by up to 65%, and a 26% reduction of disease index in *Rhizoctonia*-susceptible cultivars under field conditions using seed-priming with a tripartite bacterial consortium consisting of *Pseudomonas poae, Ps. brassicacearum*, and *Serratia plymuthica* (Wolfgang et al., 2020). On the other hand, the combination of *Pseudomonas* and *Bacillus* BCAs did not increase the biocontrol of *Globisporangium* (*Pythium*) *ultimum* in sugar beet compared to the single usage of *Pseudomonas* strains (Georgakopoulos et al., 2002). However, approaches using consortia are confronted with the hurdle that every BCA has to be licensed separately, which poses high investment costs.

#### 3.1.3. Amendments and the importance of C/N ratio for biocontrol

Indirectly managing microbial communities in field soils via amendments was practiced for millennia. With recent molecular methods, we can nowadays identify the specific responses of soil microbiomes upon different amendments on the microbial community level (Vida et al., 2020). Organic amendments can increase yield or decrease the severity of diseases caused by beet pathogens or pests (e.g., Postma and Schilder, 2015). Despite promising results, the main disadvantage of amendments scientifically is the lack of comparability due to multivariate differences, including age, composition, present microbiota, source of single components, etc. N-rich, easily decomposable amendments favor *R. solani* infection in the short term but suppress it with increasing decomposition time in lettuce (*Lactuca sativa*, L.). Furthermore, an amendment-dependent trade-off between plant growth promotion and microbial disease control capability was observed (Bonanomi et al., 2020). C/N-ratio of amendments may be an important factor for microbiome capability for pathogen control: sugar beets exude a comparably high amount of inorganic nitrogen (exudate C/N-ratio ≤ 12:1), and *R. solani* infection was controlled under laboratory conditions by locally applying 2% solutions of glucose, fructose, sorbose or xylose to germinating seedlings. This control effect was reversible by adding additional nitrogen sources to the soil (Shimizu et al., 2018). N content of the taproot may play a role in some pathogeneses: *R. solani*-tolerant cultivars often contain higher concentrations of alpha-amino N. The concentration of alpha-amino N within the root further increases during infection, irrespective of the *Rhizoctonia* tolerance of the sugar beet cultivar (Ogata et al., 2006). Still, soil microbiota species identity plays a role in disease onset: using N-rich vermicompost as an amendment, a high amount of *Firmicutes* and *Streptomycetacae* in the sugar beet root community was found (Wolfgang et al., 2020); these taxa are known to display antagonism toward *Rhizoctonia* (Table 1). Competition between pathogen and microbial communities for nitrogen sources is hereby suggested to influence biocontrol of damping-off caused by *R. solani*, but this needs further evaluation.

#### 3.1.4. Suppressive soils: a treasure chest for future biocontrol options

Disease-suppressive soils harbor microbiomes where a given phytopathogen cannot establish or does not cause disease despite its prevalence and abundance in the soil. Soil suppressiveness is lost after soil sterilization and thus attributed to specific microbiome composition or members (Mendes et al., 2011; van der Voort et al., 2016). Soil suppressiveness to *R. solani* in sugar beet was investigated elaborately, broadening our understanding of both sugar beet microbiomes and plant microbiomes in general. Soil suppressiveness may evolve under continuous cropping conditions (Raaijmakers and Mazzola, 2016), but the exact prerequisites of soil microbiomes to develop suppressiveness are yet not identified. Still, slow-growing and heat-tolerant bacteria like *Streptomycetaceae, Micrococcaceae, Mycobacteriaceae*, and *Solibacteraceae* correlate with suppressiveness toward *Rhizoctonia* (van der Voort et al., 2016).

**Figure 2:**
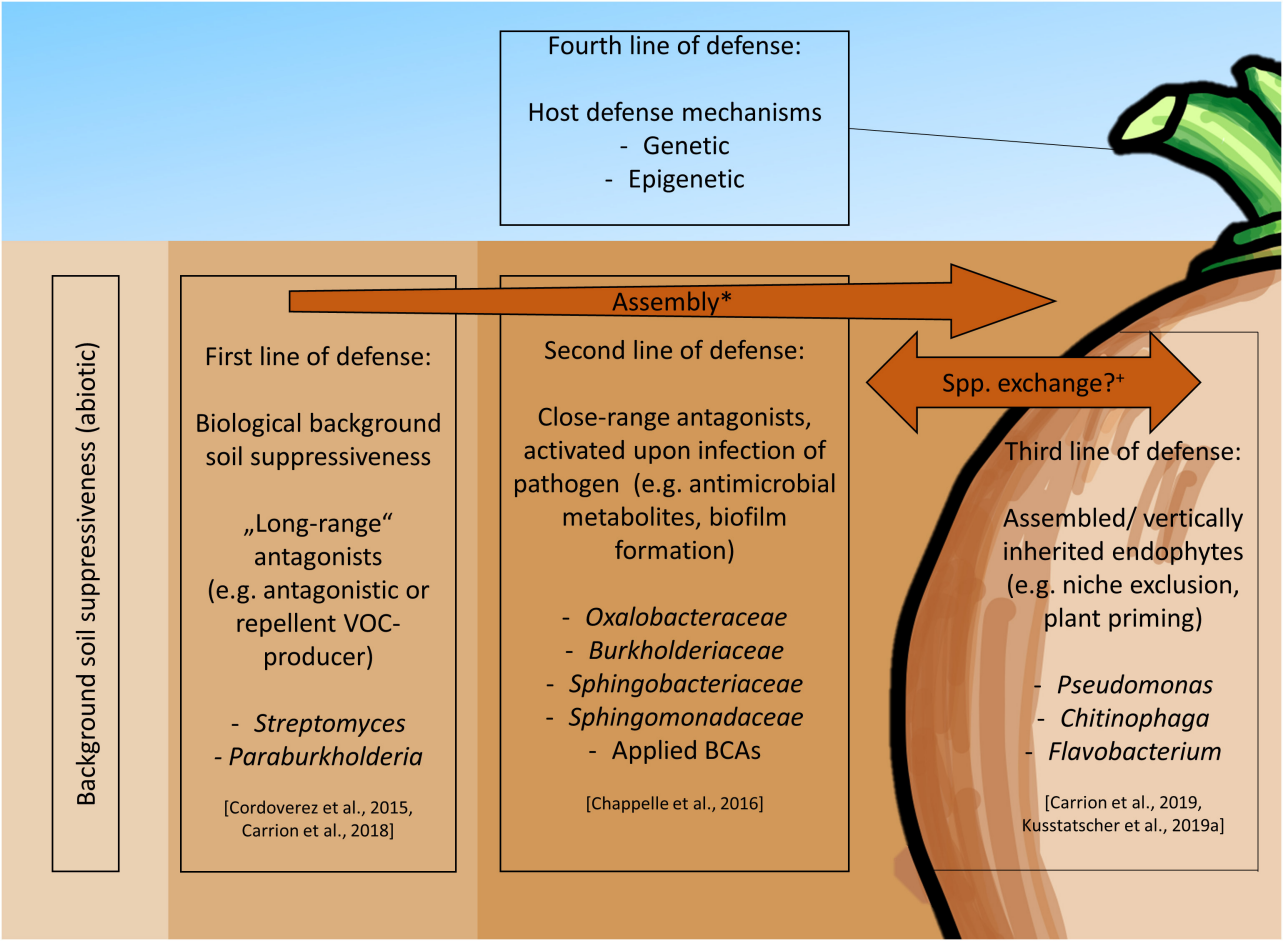
Microbiome-based defense mechanism toward *Rhizoctonia solani* in sugar beet. *Root exudate-mediated enrichment of rhizosphere-associated microbes. ^+^The deposition of initial seed endophytes from taproot toward the rhizosphere is not proven in sugar beet so far.

Several mechanisms for sugar beet protection through the activity of associated microbiota in suppressive soils were found, hereby categorized into the “four lines of defense” (Figure 2) with *R. solani* as the model pathogen. The first defensive line includes the biotic background suppressiveness of soil, for instance, mediated by microbes inhibiting pathogens via volatile organic compounds (VOC) as “long-range” antagonists, e.g., plant growth-promoting *Streptomyces* spp. and *Paraburkholderia graminis* isolates originating from sugar beet rhizosphere (Cordovez et al., 2015; Carrión et al., 2018). The second line of defense includes microbes activated upon *Rhizoctonia* infection in the rhizosphere: if *R. solani* is applied to sugar beet seedlings in suppressive soil, *Oxalobacteraceae, Burkholderiaceae, Sphingobacteriaceae*, and *Sphingomonadaceae* are significantly enriched compared to non-stressed rhizosphere (Chapelle et al., 2016). Transcriptomic analyses indicated *R. solani* to produce oxalic and phenylacetic acids, activating specific rhizobacteria. These activated bacteria show upregulated ppGpp pathways, leading to fungal growth inhibition, induced plant resistance responses, and co-activation of other microorganisms. The third line of defense includes root endophytes changing their transcriptomic profile upon *Rhizoctonia* infection, namely *Pseudomonas, Chitinophaga*, and *Flavobacterium* (Carrión et al., 2019). These bacteria may therefore be regarded as soterobionts (disease-preventing bacteria; Cernava and Berg, 2022) of the sugar beet-*Rhizoctonia* pathosystem. Lastly, the fourth line of defense contains the plant host-specific defense mechanisms and physiological traits, that as well affect the associated microbes and can be influenced via breeding.

Pathogen tolerance may occur in susceptible cultivars grown in conducive soils, and such plants are promising sources for future biocontrol candidates. Kusstatscher and colleagues (Kusstatscher et al., 2019a) investigated the bacterial and fungal rhizosphere community of pre-harvest sugar beet taproots in conducive soils, targeting *R. solani*-diseased beets and healthy beets surrounded by diseased beets. *Lactobacillales, Candida, Fusarium*, and *Penicillium* were identified as indicator species for diseased beets, while *Flavobacteriales, Cyanobacteria, Plectosphaerella, Vishniacozyma*, and *Sordariomycetes* were indicators for symptomless beets under pathogen pressure. Our current knowledge on microbiota active in disease-suppressive soils and on indicator taxa for disease tolerance in conducive soils provides the potential to predict realized pathogen pressure on a given field, as well as several disease-preventing taxa that can be applied as BCAs in future studies.

### 3.2. Controlling root rots in postharvest beets

After harvest, sugar beet taproots are usually stored in piles directly on the field or in the proximity of sugar refineries (Draycott, 2003; Kusstatscher et al., 2019b; Kohout et al., 2020). As storage time increases, autochthonous sucrose synthase of sugar beet cleaves sucrose to fructose and glucose to provide life-sustaining metabolism. In unwounded sugar beets, >90% of sucrose loss is due to the activity of autogenous sucrose synthase (Klotz and Finger, 2004). However, the combination of high water and sucrose content with wounds, cracks, or root tip breakage provides optimal conditions for microbial infections (Liebe and Varrelmann, 2016; Liebe et al., 2016; Kusstatscher et al., 2019b). Especially fungal infections with *Penicillium* spp. and *Botrytis cinerea* lead to an increase in fructose and glucose concentration, mainly attributable to fungal acid invertases (Klotz and Finger, 2004). Furthermore, oomycetes like *Aphanomyces cochlioides* DRECHSLER can cause severe sugar losses (Campbell and Klotz, 2006). Since fungi are the main group causing post-harvest sucrose losses, post-harvest microbiome studies focus—in contrast to all other sugar beet-related microbiome research—on fungal communities.

Damage to the root is the most important factor determining fungal rot severity, followed by genotype and environment (Liebe and Varrelmann, 2016). After harvest, the fungal microbiome of sugar beet taproots is dominated by *Plectosphaerella cucumerina, Pyrenochaeta*, and *Leptosphaeria*. The oomycete community is dominated by *Aphanomyces* and *Globisporangium*/*Pythium*. In addition to mechanical damage, storage temperature has severe consequences for the fungal community in stored roots: while lower temperatures (8°C) favor *Botrytis*, higher temperatures (20°C) favor *Fusarium, Penicillium*, and partially *Pichia* (Liebe and Varrelmann, 2016). During rot, the overall diversity of fungi decreases (Liebe et al., 2016; Kusstatscher et al., 2019b), but diversity within the genus *Fusarium* increases (Liebe et al., 2016). These fungal taxa however could also be targeted using biocontrol approaches, reducing fungal sucrose degradation in stored beets, to increase efficiency and thus sustainability of sugar beet production.

The microbiome in fields, where stored beets originate from, has a big influence on associated fungal taxa. Storage rots were long thought to be erratic, because visually healthy beets may develop rot symptoms (Campbell and Klotz, 2006; Christ et al., 2011). However, fungal indicator species for storability were identified in the rhizosphere and outer endosphere of harvested beets, namely *Plectosphaerella* and *Vishniacozyma*. These taxa were associated with symptomless stored beets next to rotten beets while decaying beets were characterized by a high abundance of *Lactobacillus, Gluconobacter, Candida*, and *Penicillium* (Kusstatscher et al., 2019b). Measuring such indicator taxa in field soils before storage would enable refineries to rank and process sugar beet lots based on their proposed storability, reducing losses due to storage rots.

### 3.3. Microbiome management for healthy sugar beet production and storage

**Figure 3:**
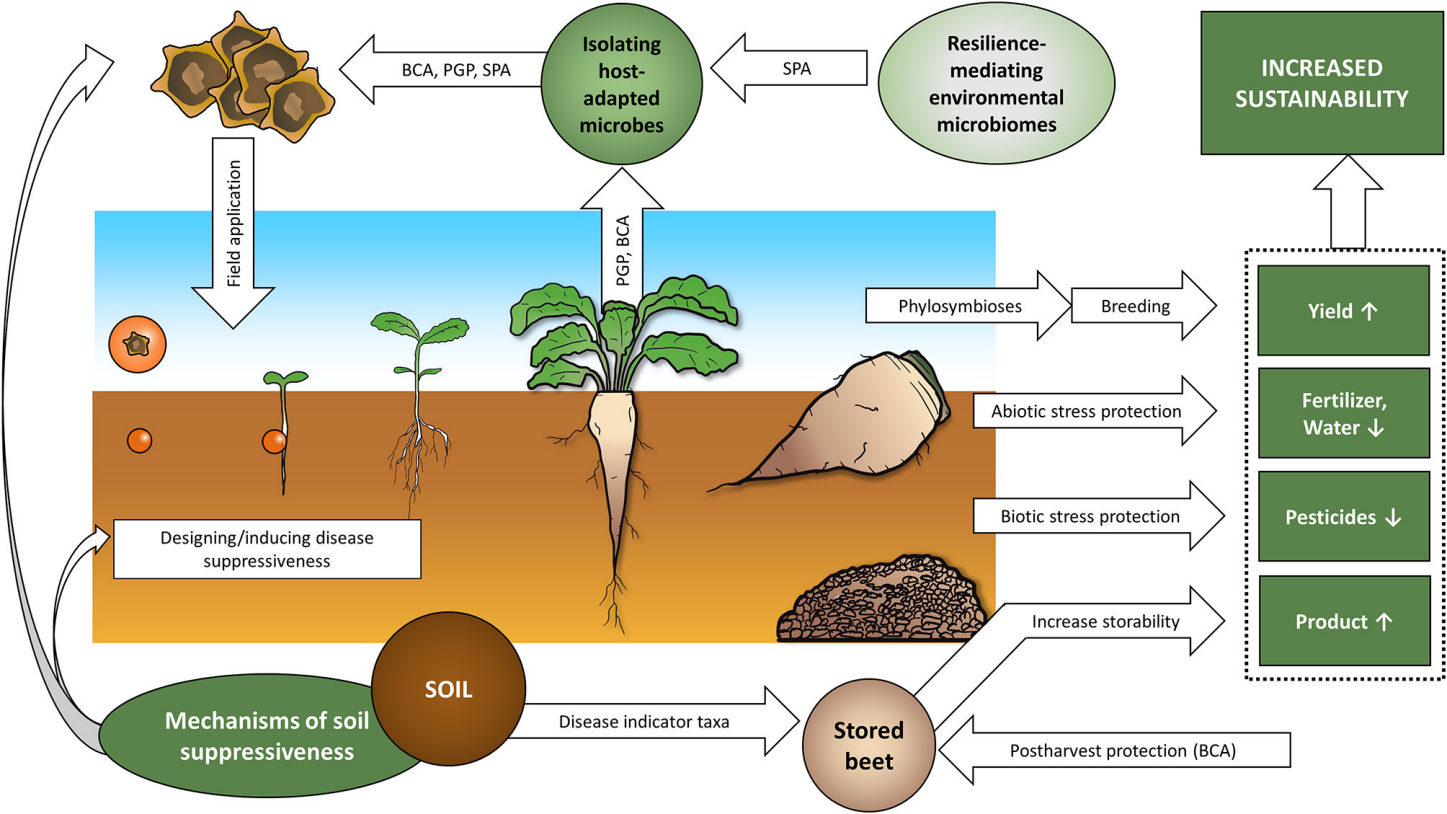
Summary of microbiome-based targets to increase sugar beet farming sustainability. BCA, Biological control agents; PGP, plant growth promoter; SPA, stress protecting agents. For a description see Section 3.3.

Microbiome-based management or modulation options for all life stages of the sugar beet were already suggested and partially commercialized (Figure 3). Sugar beet-adapted microbes can be isolated to test antagonism toward pathogens and plant growth-promoting traits. Microbes derived from extreme environments can be investigated for tolerance-mediating effects to mitigate abiotic stress. Protection from biotic and abiotic stressors is especially important in the vulnerable seedling state during rhizosphere establishment, therefore seed priming technologies are specifically important to assist in the assembly of beneficial rhizosphere microbiomes. With microbial abiotic and biotic stress protection, a reduction of fertilizers and pesticides is possible. Investigating suppressive soils provides the opportunity to directly (application of transplants or suppressiveness-mediating microbiota) or indirectly (modulation via amendments) modulate soil microbiota to the benefit of crop vigor. Phylosymbioses studies could be used for microbiome-assisted breeding, potentially further increasing yield or desired crop traits. Finally, considering disease indicator taxa and biocontrol approaches in postharvest beets may increase storability, consequently efficiency and ecological sustainability of sugar beet farming. In this way, microbiome-based management systems provide tools to meet the upcoming agricultural challenges we will face in the Anthropocene (Berg and Cernava, 2022).

## 4. Conclusions and outlook

1. Sugar beet is a very interesting model plant for microbiome research due to its recent and documented breeding history, narrow genetic background, economic importance, well-studied relatives, susceptibility toward fungal pathogens, and also its long history of microbiological research.
2. The well-studied sugar beet microbiome is associated with some breakthroughs in plant microbiome research. However, additional holistic and especially mechanistic studies using comprehensive combinations of advanced microbiome research methods are required. Further research should specifically consider the known spatial and temporal scales realized in the sugar beet holobiont, being summarized in this review. Therefore, especially sampling design and procedures should be described in as much detail as possible (age, life stage, cultivar, plant organ, position on plant organ, etc.) to properly contextualize observations and to increase comparability between different studies.
3. Future research should as well address not only bacteria, but all microbiome members, including protists and viruses to provide a more holistic view of potential autochthonous regulatory mechanisms and interactions in a given microbiome. Combined studies of the plant genome and microbiome by multi-omics suggest further ground-breaking results.
4. Sugar beet microbiomes were strongly shaped by breeding, and early evidence for plant-microbe coevolution was found. Phylosymbiosis studies will promote microbiome-assisted breeding efforts, which is essential for further sustainable sugar beet production.
5. A system-immanent challenge for scientific work is the constant success and development in sugar beet breeding, together with the characteristic mating system. Modern sugar beet cultivars are hybrids, new cultivars are bred and licensed every year and usually have to be licensed on a national level. Consequently, most scientific studies use different cultivars, because of availability in a given country or shutdown of production. In this way, sugar beet microbiome research always has to consider cultivar-dependent effects as an additional layer of variability when comparing different studies.
6. The sugar beet microbiome is highly diverse, microhabitat- and cultivar-specific. Although several successful microbiome management strategies were already developed, understanding plant-microbiome interactions novel microbiome management strategies. In particular, the connection between leaf microbiomes and susceptibility toward air-borne foliage diseases (e.g., diseases caused by *Ramularia beticola, Erysiphe betae*, or *Cercospora beticola*) or insect pests are potential targets for future biocontrol strategies. Moreover, adapting sugar beet cultivation to abiotic stress conditions under climate change, and reducing greenhouse gas emissions are important challenges of the future.

## Author contributions

AW and GB wrote the manuscript and created figures. All authors contributed to the article and approved the submitted version.

## References

[B1] Abdelfattah A. Wisniewski M. Schena L. Tack A. J. M. (2021). Experimental evidence of microbial inheritance in plants and transmission routes from seed to phyllosphere and root. Environ. Microbiol. 23, 2199–2214. 10.1111/1462-2920.15392 33427409

[B2] Allen M. F. Boosalis M. G. Kerr E. D. Muldoon A. E. Larsen H. J. (1985). Population dynamics of sugar beets, Rhizoctonia solani, and Laetisaria arvalis: Responses of a host, plant pathogen, and hyperparasite to perturbation in the field. Appl. Environ. Microbiol. 50, 1123–1127. 10.1128/aem.50.5.1123-1127.1985 16346925 PMC238710

[B3] Andersen J. B. Koch B. Nielsen T. H. Sørensen D. Hansen M. Nybroe O.. (2003). Surface motility in *Pseudomonas sp*. DSS73 is required for efficient biological containment of the root-pathogenic microfungi Rhizoctonia solani and Pythium ultimum. Microbiology. 149, 37–46. 10.1099/mic.0.25859-0 12576578

[B4] Badri D. V. Vivanco J. M. (2009). Regulation and function of root exudates. Plant, Cell Environ. 32, 666–681. 10.1111/j.1365-3040.2009.01926.x 19143988

[B5] Bais H. P. Weir T. L. Perry L. G. Gilroy S. Vivanco J. M. (2006). The role of root exudates in rhizosphere interactions with plants and other organisms. Annu. Rev. Plant Biol. 57, 233–266. 10.1146/annurev.arplant.57.032905.105159 16669762

[B6] Bargabus R. L. Zidack N. K. Sherwood J. E. Jacobsen B. J. (2002). Characterisation of systemic resistance in sugar beet elicited by a non-pathogenic, phyllosphere-colonizing *Bacillus mycoides*, biological control agent. Physiol. Mol. Plant Pathol. 61, 289–298. 10.1006/pmpp.2003.0443

[B7] Bargabus R. L. Zidack N. K. Sherwood J. E. Jacobsen B. J. (2003). Oxidative burst elicited by *Bacillus mycoides* isolate Bac J, a biological control agent, occurs independently of hypersensitive cell death in sugar beet. Mol. Plant-Microbe Interact. 16, 1145–1153. 10.1094/MPMI.2003.16.12.1145 14651348

[B8] Bargabus R. L. Zidack N. K. Sherwood J. E. Jacobsen B. J. (2004). Screening for the identification of potential biological control agents that induce systemic acquired resistance in sugar beet. Biol. Control. 30, 342–350. 10.1016/j.biocontrol.2003.11.005

[B9] Barret M. Briand M. Bonneau S. Préveaux A. Valière S. Bouchez O.. (2015). Emergence shapes the structure of the seed microbiota. Appl. Environ. Microbiol. 81, 1257–1266. 10.1128/AEM.03722-14 25501471 PMC4309697

[B10] Bashir I. War A. F. Rafiq I. Reshi Z. A. Rashid I. Shouche Y. S. (2022). Phyllosphere microbiome: Diversity and functions. Microbiol. Res. 254, 126888. 10.1016/j.micres.2021.126888 34700185

[B11] Bazany K. E. Wang J. T. Delgado-Baquerizo M. Singh B. K. Trivedi P. (2022). Water deficit affects inter-kingdom microbial connections in plant rhizosphere. Environ. Microbiol. 24, 3722–3734. 10.1111/1462-2920.16031 35582745 PMC9545320

[B12] Berendsen R. L. Pieterse C. M. J. Bakker P. A. H. M. (2012). The rhizosphere microbiome and plant health. Trends Plant Sci. 17, 478–486. 10.1016/j.tplants.2012.04.001 22564542

[B13] Berg G. Cernava T. (2022). The plant microbiota signature of the Anthropocene as a challenge for microbiome research. Microbiome 10, 1–12. 10.1186/s40168-021-01224-5 35346369 PMC8959079

[B14] Berg G. Köberl M. Rybakova D. Müller H. Grosch R. Smalla K. (2017). Plant microbial diversity is suggested as the key to future biocontrol and health trends. FEMS Microbiol. Ecol. 93, 1–9. 10.1093/femsec/fix050 28430944

[B15] Berg G. Krause R. Mendes R. (2015). Cross-kingdom similarities in microbiome ecology and biocontrol of pathogens. Front. Microbiol. 6, 1–5. 10.3389/fmicb.2015.01311 26635772 PMC4658424

[B16] Berg G. Kusstatscher P. Abdelfattah A. Cernava T. Smalla K. (2021). Microbiome modulation—toward a better understanding of plant microbiome response to microbial inoculants. Front. Microbiol. 12, 1–12. 10.3389/fmicb.2021.650610 33897663 PMC8060476

[B17] Berg G. Raaijmakers J. M. (2018). Saving seed microbiomes. ISME J. 12, 1167–1170. 10.1038/s41396-017-0028-2 29335636 PMC5931960

[B18] Berg G. Rybakova D. Fischer D. Cernava T. Vergès M. C. C. Charles T.. (2020). Microbiome definition re-visited: old concepts and new challenges. Microbiome 8, 1–22. 10.1186/s40168-020-00875-0 32605663 PMC7329523

[B19] Berg G. Smalla K. (2009). Plant species and soil type cooperatively shape the structure and function of microbial communities in the rhizosphere. FEMS Microbiol. Ecol. 68, 1–13. 10.1111/j.1574-6941.2009.00654.x 19243436

[B20] Bergna A. Cernava T. Rändler M. Grosch R. Zachow C. Berg G. (2018). Tomato seeds preferably transmit plant beneficial endophytes. Phytobiomes. 2, 183–193. 10.1094/PBIOMES-06-18-0029-R

[B21] Bertoldo G. Della Lucia M. C. Squartini A. Concheri G. Broccanello C. Romano A.. (2021). Endophytic microbiome responses to sulfur availability in Beta vulgaris (L.). Int. J. Mol. Sci. 22. 10.3390/ijms22137184 34281236 PMC8269133

[B22] Bonanomi G. Zotti M. Idbella M. Di Silverio N. Carrino L. Cesarano G.. (2020). Decomposition and organic amendments chemistry explain contrasting effects on plant growth promotion and suppression of Rhizoctonia solani damping off. PLoS ONE. 15, 1–20. 10.1371/journal.pone.0230925 32271811 PMC7144968

[B23] Bosemark N. O. (1979). “Genetic poverty of the sugarbeet in Europe,” in Broadening the Genetic Base of Crops, Zeven A. van Harten A. M. (eds). (Wageningen: Centre for Agricultural Publishing and Documentation) p. 28–35.

[B24] Broccanello C. Ravi S. Deb S. Bolton M. Secor G. Richards C.. (2022). Bacterial endophytes as indicators of susceptibility to Cercospora leaf spot (CLS) disease in Beta vulgaris L. Sci. Rep. 12, 1–13. 10.1038/s41598-022-14769-8 35739218 PMC9226160

[B25] Büttner G. Pfähler B. Märländer B. (2004). Greenhouse and field techniques for testing sugar beet for resistance to Rhizoctonia root and crown rot. Plant Breed. 123, 158–166. 10.1046/j.1439-0523.2003.00967.x

[B26] Cai D. Kleine M. Kifle S. Harloff H. J. Sandal N. N. Marcker K. A.. (1997). Positional cloning of a gene for nematode resistance in sugar beet. Science (80-.). 275, 832–834. 10.1126/science.275.5301.832 9012350

[B27] Çakmakçi R. Kantar F. Sahin F. (2001). Effect of N_2_-fixing bacterial inoculations on yield of sugar beet and barley. J. Plant Nutr. Soil Sci. 164, 527–531. 10.1002/1522-2624(200110)164:5<527::AID-JPLN527>3.0.CO;2-1

[B28] Campbell L. G. Klotz K. (2006). “Storage,” in Sugar Beet, Draycott A. P. (ed). (Oxford: Blackwell Publishing Ltd) p. 387–408. 10.1002/9780470751114.ch15

[B29] Cardinale M. Ratering S. Sadeghi A. Pokhrel S. Honermeier B. Schnell S. (2020). The response of the soil microbiota to long-term mineral and organic nitrogen fertilization is stronger in the bulk soil than in the rhizosphere. Genes (Basel). 11, 456. 10.3390/genes11040456 32331348 PMC7230438

[B30] Carrión V. J. Cordovez V. Tyc O. Etalo D. W. de Bruijn I. de Jager V. C. L.. (2018). Involvement of Burkholderiaceae and sulfurous volatiles in disease-suppressive soils. ISME J. 12, 2307–2321. 10.1038/s41396-018-0186-x 29899517 PMC6092406

[B31] Carrión V. J. Perez-Jaramillo J. Cordovez V. Tracanna V. De Hollander M. Ruiz-Buck D.. (2019). Pathogen-induced activation of disease-suppressive functions in the endophytic root microbiome. Science (80-.). 366, 606–612. 10.1126/science.aaw9285 31672892

[B32] Cernava T. Berg G. (2022). The emergence of disease-preventing bacteria within the plant microbiota. Environ. Microbiol. 24, 3259–3263. 10.1111/1462-2920.15896 35001485 PMC9541158

[B33] Chapelle E. Mendes R. Bakker P. A. H. Raaijmakers J. M. (2016). Fungal invasion of the rhizosphere microbiome. ISME J. 10, 265–268. 10.1038/ismej.2015.82 26023875 PMC4681858

[B34] Christ D. S. Märländer B. Varrelmann M. (2011). Characterization and mycotoxigenic potential of Fusarium species in freshly harvested and stored sugar beet in Europe. Phytopathology. 101, 1330–1337. 10.1094/PHYTO-01-11-0002 21770776

[B35] Compant S. Cambon M. C. Vacher C. Mitter B. Samad A. Sessitsch A. (2021). The plant endosphere world–bacterial life within plants. Environ. Microbiol. 23, 1812–1829. 10.1111/1462-2920.15240 32955144

[B36] Compant S. Reiter B. Sessitsch A. Nowak J. Clément C. Barka E. A. (2005). Endophytic colonization of Vitis vinifera L. by plant growth-promoting bacterium Burkholderia sp. strain PsJN. Appl. Environ. Microbiol. 71, 1685–1693. 10.1128/AEM.71.4.1685-1693.2005 15811990 PMC1082517

[B37] Cook R. J. Thomashow L. S. Weller D. M. Fujimoto D. Mazzola M. Bangera G.. (1995). Molecular mechanisms of defense by rhizobacteria against root disease. Proc. Natl. Acad. Sci. U. S. A. 92, 4197–4201. 10.1073/pnas.92.10.4197 11607544 PMC41910

[B38] Cordovez V. Carrion V. J. Etalo D. W. Mumm R. Zhu H. van Wezel G. P.. (2015). Diversity and functions of volatile organic compounds produced by Streptomyces from a disease-suppressive soil. Front. Microbiol. 6, 1–13. 10.3389/fmicb.2015.01081 26500626 PMC4598592

[B39] Cui R. Geng G. Wang G. Stevanato P. Dong Y. Li T.. (2022). The response of sugar beet rhizosphere micro-ecological environment to continuous cropping. Front. Microbiol. 13. 10.3389/fmicb.2022.956785 36160206 PMC9490479

[B40] Darmency H. Klein E. K. Gestat De Garanb,é T. Gouyon P. H. Richard-Molard M. Muchembled C. (2009). Pollen dispersal in sugar beet production fields. Theor. Appl. Genet. 118, 1083–1092. 10.1007/s00122-009-0964-y 19183859

[B41] de la Fuente Cant,ó C. Simonin M. King E. Moulin L. Bennett M. J. Castrillo G.. (2020). An extended root phenotype: the rhizosphere, its formation and impacts on plant fitness. Plant J. 103, 951–964. 10.1111/tpj.14781 32324287

[B42] Della Lucia M. C. Bertoldo G. Broccanello C. Maretto L. Ravi S. Marinello F.. (2021). Novel effects of leonardite-based applications on sugar beet. Front. Plant Sci. 12, 1–10. 10.3389/fpls.2021.646025 33815453 PMC8013720

[B43] Dent K. C. Stephen J. R. Finch-Savage W. E. (2004). Molecular profiling of microbial communities associated with seeds of Beta vulgaris subsp. vulgaris (sugar beet). J. Microbiol. Methods 56, 17–26. 10.1016/j.mimet.2003.09.001 14706747

[B44] Desoignies N. Schramme F. Ongena M. Legrève A. (2013). Systemic resistance induced by Bacillus lipopeptides in beta vulgaris reduces infection by the rhizomania disease vector Polymyxa betae. Mol. Plant Pathol. 14, 416–421. 10.1111/mpp.12008 23279057 PMC6638685

[B45] Dohm J. C. Minoche A. E. Holtgräwe D. Capella-Gutiérrez S. Zakrzewski F. Tafer H.. (2014). The genome of the recently domesticated crop plant sugar beet (Beta vulgaris). Nature. 505, 546–549. 10.1038/nature12817 24352233

[B46] Dong Z. Canny M. J. McCully M. E. Roboredo M. R. Cabadilla C. F. Ortega E.. (1994). A nitrogen-fixing endophyte of sugarcane stems: a new role for the apoplast. Plant Physiol. 105, 1139–1147. 10.1104/pp.105.4.1139 12232271 PMC159442

[B47] Draycott A. P. (2003). “Introduction,” in Nutrients for sugarbeet production: soil-plant relationships, Draycott A. P. Christenson D. R. (Wallingford, UK: CAB International) p. 1–8. 10.1079/9780851996233.0001

[B48] Du J. Song B. Jia Q. Liu S. Li X. Liu H.. (2022a). Effect of long-term sugar beet cultivation on rhizosphere bacterial diversity, community structure and sugar yield of sugar beet. Rhizosphere. 22, 100507. 10.1016/j.rhisph.2022.100507

[B49] Du J. Song B. Li X. Huang W. (2022b). Long-term cultivation of sugar beet: Effect on rhizosphere micro-flora, soil fertility and beet productivity. Sugar Tech. 24, 1821–1831. 10.1007/s12355-022-01124-4

[B50] Dunne C. Moenne-Loccoz Y. de Bruijn F. J. O'Gara F. (2000). Overproduction of an inducible extracellular serine protease improves biological control of Pythium ultimum by Stenotrophomonas maltophilia strain W81. Microbiology. 146, 2069–2078. 10.1099/00221287-146-8-2069 10931911

[B51] Eberlein C. Heuer H. Westphal A. (2020). Biological suppression of populations of Heterodera schachtii adapted to different host genotypes of sugar beet. Front. Plant Sci. 11, 1–13. 10.3389/fpls.2020.00812 32636857 PMC7317003

[B52] Ellis R. J. Thompson I. P. Bailey M. J. (1999). Temporal fluctuations in the pseudomonad population associated with sugar beet leaves. FEMS Microbiol. Ecol. 28, 345–356. 10.1111/j.1574-6941.1999.tb00589.x

[B53] El-Tarabily K. A. (2004). Suppression of *Rhizoctonia solani* diseases of sugar beet by antagonistic and plant growth-promoting yeasts. J. Appl. Microbiol. 96, 69–75. 10.1046/j.1365-2672.2003.02043.x 14678160

[B54] Errakhi R. Lebrihi A. Barakate M. (2009). *In vitro* and *in vivo* antagonism of actinomycetes isolated from Moroccan rhizospherical soils against Sclerotium rolfsii: A causal agent of root rot on sugar beet (Beta vulgaris L.). J. Appl. Microbiol. 107, 672–681. 10.1111/j.1365-2672.2009.04232.x 19302305

[B55] FAOSTAT (2022). Available online at: https://www.fao.org/faostat/en/#data (accessed June 24, 2022).

[B56] Farhaoui A. Adadi A. Tahiri A. El Alami N. Khayi S. Mentag R.. (2022). Biocontrol potential of plant growth-promoting rhizobacteria (PGPR) against *Sclerotiorum rolfsii* diseases on sugar beet (Beta vulgaris L.). *Physiol. Mol. Plant Pathol*. 119, 101829. 10.1016/j.pmpp.2022.101829

[B57] Fischer H. E. (1989). Origin of the “Weisse Schlesische Rübe” (white Silesian beet) and resynthesis of sugar beet. Euphytica. 41, 75–80. 10.1007/BF00022414

[B58] Fort T. Pauvert C. Zanne A. E. Ovaskainen O. Caignard T. Barret M.. (2021). Maternal effects shape the seed mycobiome in Quercus petraea. New Phytol. 230, 1594–1608. 10.1111/nph.17153 33341934

[B59] Francis S. A. (2006). “Development of sugar beet,” in Sugar Beet, Draycott A. P. (Oxford: Blackwell Publishing Ltd) p. 9–29. 10.1002/9780470751114.ch2

[B60] Free J. B. Williams I. Longden P. C. Johnson M. G. (1975). Insect pollination of sugar-beet (Beta vulgaris) seed crops. Ann. Appl. Biol. 81, 121–134. 10.1111/j.1744-7348.1975.tb00529.x34292667

[B61] Galewski P. Funk A. McGrath J. M. (2022). Select and sequence of a segregating sugar beet population provides genomic perspective of host resistance to seedling Rhizoctonia solani Infection. Front. Plant Sci. 12, 785267. 10.3389/fpls.2021.785267 35095959 PMC8793884

[B62] Galewski P. McGrath J. M. (2020). Genetic diversity among cultivated beets (Beta vulgaris) assessed via population-based whole genome sequences. BMC Genomics. 21, 1–14. 10.1186/s12864-020-6451-1 32122300 PMC7053042

[B63] Gasser I. Cardinale M. Müller H. Heller S. Eberl L. Lindenkamp N.. (2011). Analysis of the endophytic lifestyle and plant growth promotion of Burkholderia terricola ZR2-12. Plant Soil. 347, 125–136. 10.1007/s11104-011-0833-8

[B64] Gasser I. Müller H. Berg G. (2009). Ecology and characterization of polyhydroxyalkanoate-producing microorganisms on and in plants. FEMS Microbiol. Ecol. 70, 142–150. 10.1111/j.1574-6941.2009.00734.x 19656194

[B65] Georgakopoulos D. G. Fiddaman P. Leifert C. Malathrakis N. E. (2002). Biological control of cucumber and sugar beet damping-off caused by Pythium ultimum with bacterial and fungal antagonists. J. Appl. Microbiol. 92, 1078–1086. 10.1046/j.1365-2672.2002.01658.x 12010548

[B66] Germida J. J. Siciliano S. D. (2001). Taxonomic diversity of bacteria associated with the roots of modern, recent and ancient wheat cultivars. Biol. Fertil. Soils 33, 410–415. 10.1007/s003740100343

[B67] Gopal M. Gupta A. (2016). Microbiome selection could spur next-generation plant breeding strategies. Front. Microbiol. 7, 1–10. 10.3389/fmicb.2016.01971 28003808 PMC5141590

[B68] Gouka L. Raaijmakers J. M. Cordovez V. (2022). Ecology and functional potential of phyllosphere yeasts. Trends Plant Sci. 27, 1109–1123. 10.1016/j.tplants.2022.06.007 35842340

[B69] Grimme E. Zidack N. K. Sikora R. A. Strobel G. A. Jacobsen B. J. (2007). Comparison of *Muscodor albus* volatiles with a biorational mixture for control of seedling diseases of sugar beet and root-knot nematode on tomato. Plant Dis. 91, 220–225. 10.1094/PDIS-91-2-0220 30781008

[B70] Haj Nuaima R. Ashrafi S. Maier W. Heuer H. (2021). Fungi isolated from cysts of the beet cyst nematode parasitized its eggs and counterbalanced root damages. J. Pest Sci. 94, 563–572. 10.1007/s10340-020-01254-2

[B71] Hallmann J. Berg G. (2007). Spectrum and population dynamics of bacterial root endophytes. Microb. Root Endophytes 9, 15–31. 10.1007/3-540-33526-9_2

[B72] Haque M. E. Lakshman D. K. Qi A. Khan M. F. R. (2021). Penicillium pinophilum has the potential to reduce damping-off caused by *Rhizoctonia solani* in sugar beet. Sugar Tech. 23, 872–880. 10.1007/s12355-021-00958-8

[B73] Hardoim P. R. Hardoim C. C. P. van Overbeek L. S. van Elsas J. D. (2012). Dynamics of seed-borne rice endophytes on early plant growth stages. PLoS ONE. 7, e30438. 10.1371/journal.pone.0030438 22363438 PMC3281832

[B74] Hardoim P. R. van Overbeek L. S. Berg G. Pirttil,ä A. M. Compant S. Campisano A.. (2015). The hidden world within plants: ecological and evolutionary considerations for defining functioning of microbial endophytes. Microbiol. Mol. Biol. Rev. 79, 293–320. 10.1128/MMBR.00050-14 26136581 PMC4488371

[B75] Hegarty T. W. (1977). Seed and seedling susceptibility to phased moisture stress in soil. J. Exp. Bot. 28, 659–668. 10.1093/jxb/28.3.659

[B76] Hergert G. W. (2010). Sugar beet fertilization. Sugar Tech. 12, 256–266. 10.1007/s12355-010-0037-1

[B77] Hoffmann C. Loel J. (2015). Bedeutung der Züchtung für den Ertragsanstieg von Zuckerrüben. Sugar Ind. 140, 48–56. 10.36961/si16195

[B78] Hoffmann C. M. Kenter C. (2018). Yield potential of sugar beet–have we hit the ceiling? Front. Plant Sci. 9, 1–6. 10.3389/fpls.2018.00289 29599787 PMC5863500

[B79] Hoffmann C. M. Kenter C. Bloch D. (2005). Marc concentration of sugar beet (Beta vulgaris L) in relation to sucrose storage. J. Sci. Food Agric. 85, 459–465. 10.1002/jsfa.2002

[B80] Houlden A. Timms-Wilson T. M. Day M. J. Bailey M. J. (2008). Influence of plant developmental stage on microbial community structure and activity in the rhizosphere of three field crops. FEMS Microbiol. Ecol. 65, 193–201. 10.1111/j.1574-6941.2008.00535.x 18616582

[B81] Huang W. Sun D. Fu J. Zhao H. Wang R. An Y. (2020). Effects of continuous sugar beet cropping on rhizospheric microbial communities. Genes (Basel). 11, 13. 10.3390/genes11010013 31877827 PMC7017100

[B82] Huang W. Sun D. Wang R. An Y. (2021). Integration of transcriptomics and metabolomics reveals the responses of sugar beet to continuous cropping obstacle. Front. Plant Sci. 12, 1–11. 10.3389/fpls.2021.711333 34777408 PMC8578061

[B83] Huang Y. Kuang Z. Wang W. Cao L. (2016). Exploring potential bacterial and fungal biocontrol agents transmitted from seeds to sprouts of wheat. Biol. Control. 98, 27–33. 10.1016/j.biocontrol.2016.02.013

[B84] Ikeda S. Okazaki K. Takahashi H. Tsurumaru H. Minamisawa K. (2023). Seasonal shifts in bacterial community structures in the lateral root of sugar beet grown in an andosol field in Japan. Microbes Environ. 38, 1–13. 10.1264/jsme2.ME22071 36754423 PMC10037095

[B85] Islam M. T. Hashidoko Y. Deora A. Ito T. Tahara S. (2005). Suppression of damping-off disease in host plants by the rhizoplane bacterium *Lysobacter sp*. strain SB-K88 is linked to plant colonization and antibiosis against soilborne Peronosporomycetes. Appl. Environ. Microbiol. 71, 3786–3796. 10.1128/AEM.71.7.3786-3796.2005 16000790 PMC1169021

[B86] Jacobs M. J. Bugbee W. M. Gabrielson D. A. (1985). Enumeration, location, and characterization of endophytic bacteria within sugar beet roots. Can. J. Bot. 63, 1262–1265. 10.1139/b85-174

[B87] Jacobsen B. J. (2006). Root rot diseases of sugar beet. Proc. Nat. Sci. Matica Srp. Novi Sad. 2006, 9–19. 10.2298/ZMSPN0610009J

[B88] Jaggard K. W. Qi A. Semenov M. A. (2007). The impact of climate change on sugarbeet yield in the UK: 1976–2004. J. Agric. Sci. 145, 367–375. 10.1017/S0021859607006922

[B89] Jakubíková L. Šubíková V. Nemčovi,č M. Farkaš V. (2006). Selection of natural isolates of Trichoderma spp. for biocontrol of Polymyxa betae as a vector of virus causing rhizomania in sugar beet. Biologia (Bratisl). 61, 347–351. 10.2478/s11756-006-0063-3

[B90] James E. K. Gyaneshwar P. Mathan N. Barraquio W. L. Reddy P. M. Iannetta P. P. M.. (2002). Infection and colonization of rice seedlings by the plant growth-promoting bacterium Herbaspirillum seropedicae Z67. Mol. Plant-Microbe Interact. 15, 894–906. 10.1094/MPMI.2002.15.9.894 12236596

[B91] Jammer A. Albacete A. Schulz B. Koch W. Weltmeier F. van der Graaff E.. (2020). Early-stage sugar beet taproot development is characterized by three distinct physiological phases. Plant Direct. 4, 1–29. 10.1002/pld3.221 32766510 PMC7395582

[B92] Johnston-Monje D. Lundberg D. S. Lazarovits G. Reis V. M. Raizada M. N. (2016). Bacterial populations in juvenile maize rhizospheres originate from both seed and soil. Plant Soil. 405, 337–355. 10.1007/s11104-016-2826-0

[B93] Johnston-Monje D. Raizada M. N. (2011). Conservation and diversity of seed associated endophytes in Zea across boundaries of evolution, ethnography and ecology. PLoS ONE. 6, e20396. 10.1371/journal.pone.0020396 21673982 PMC3108599

[B94] Johnston-Monje D. Raizada M. N. (2013). Surveying diverse zea seed for populations of bacterial endophytes. Mol. Microb. Ecol. Rhizosph. 1, 445–455. 10.1002/9781118297674.ch42

[B95] Kaga H. Mano H. Tanaka F. Watanabe A. Kaneko S. Morisaki H. (2009). Rice seeds as sources of endophytic bacteria. Microbes Environ. 24, 154–162. 10.1264/jsme2.ME09113 21566368

[B96] Karimi E. Sadeghi A. Dehaji P. A. Dalvand Y. Omidvari M. Kakuei Nezhad M. (2012). Biocontrol activity of salt tolerant Streptomyces isolates against phytopathogens causing root rot of sugar beet. Biocontrol Sci. Technol. 22, 333–349. 10.1080/09583157.2012.658552

[B97] Karimi E. Safaie N. Shams-Baksh M. Mahmoudi B. (2016). Bacillus amyloliquefaciens SB14 from rhizosphere alleviates Rhizoctonia damping-off disease on sugar beet. Microbiol. Res. 192, 221–230. 10.1016/j.micres.2016.06.011 27664740

[B98] Khorassani R. Hettwer U. Ratzinger A. Steingrobe B. Karlovsky P. Claassen N. (2011). Citramalic acid and salicylic acid in sugar beet root exudates solubilize soil phosphorus. BMC Plant Biol. 11. 10.1186/1471-2229-11-121 21871058 PMC3176199

[B99] Klaedtke S. Jacques M. A. Raggi L. Préveaux A. Bonneau S. Negri V.. (2016). Terroir is a key driver of seed-associated microbial assemblages. Environ. Microbiol. 18, 1792–1804. 10.1111/1462-2920.12977 26171841

[B100] Klotz K. L. Finger F. L. (2004). Impact of temperature, length of storage and postharvest disease on sucrose catabolism in sugarbeet. Postharvest Biol. Technol. 34, 1–9. 10.1016/j.postharvbio.2004.05.016

[B101] Kobayashi D. Y. Reedy R. M. Palumbo J. D. Zhou J. M. Yuen G. Y. (2005). A clp gene homologue belonging to the Crp gene family globally regulates lytic enzyme production, antimicrobial activity, and biological control activity expressed by Lysobacter enzymogenes strain C3. Appl. Environ. Microbiol. 71, 261–269. 10.1128/AEM.71.1.261-269.2005 15640196 PMC544266

[B102] Kohout C. K. Ukowitz C. Reiter D. Zitz U. Moder K. Emerstorfer F.. (2020). Bacterial growth dynamics and corresponding metabolite levels in the extraction area of an Austrian sugar beet factory using antimicrobial treatment. J. Sci. Food Agric. 100, 2713–2721. 10.1002/jsfa.10303 32002998

[B103] Kõiv V. Arbo K. Maiväli Ü. Kisand V. Roosaare M. Remm M.. (2019). Endophytic bacterial communities in peels and pulp of five root vegetables. PLoS ONE. 14, 1–17. 10.1371/journal.pone.0210542 30633764 PMC6329509

[B104] Koskella B. (2020). The phyllosphere. Curr. Biol. 30, R1143–R1146. 10.1016/j.cub.2020.07.037 33022257

[B105] Kusstatscher P. Cernava T. Harms K. Maier J. Eigner H. Berg G.. (2019a). Disease incidence in sugar beet fields is correlated with microbial diversity and distinct biological markers. Phytobiomes J. 3, 22–30. 10.1094/PBIOMES-01-19-0008-R

[B106] Kusstatscher P. Wicaksono W. A. Bergna A. Cernava T. Bergau N. Tissier A.. (2020). Trichomes form genotype-specific microbial hotspots in the phyllosphere of tomato. Environ. Microbiomes 15, 1–10. 10.1186/s40793-020-00364-9 33902724 PMC8067393

[B107] Kusstatscher P. Zachow C. Harms K. Maier J. Eigner H. Berg G.. (2019b). Microbiome-driven identification of microbial indicators for postharvest diseases of sugar beets. Microbiome 7, 1–12. 10.1186/s40168-019-0728-0 31391094 PMC6686572

[B108] Lange W. Brandenburg W. A. De Bock T. S. M. (1999). Taxonomy and cultonomy of beet (Beta vulgaris L.). Bot. J. Linn. Soc. 130, 81–96. 10.1111/j.1095-8339.1999.tb00785.x

[B109] Larran S. Mónaco C. Alippi H. E. (2000). Endophytic fungi in beet (Beta vulgaris var. esculenta L.) leaves. Adv. Hort. Sci. 14, 193–196. 10.1400/14064

[B110] Lee S. Y. (1996). Bacterial polyhydroxyalkanoates. Biotechnol. Bioeng. 49, 1–14.3. 10.1002/(SICI)1097-0290(19960105)49:1<1::AID-BIT1>3.0.CO;2-P 18623547

[B111] Liebe S. Varrelmann M. (2016). Effect of environment and sugar beet genotype on root rot development and pathogen profile during storage. Phytopathology. 106, 65–75. 10.1094/PHYTO-07-15-0172-R 26474333

[B112] Liebe S. Wibberg D. Winkler A. Pühler A. Schlüter A. Varrelmann M. (2016). Taxonomic analysis of the microbial community in stored sugar beets using high-throughput sequencing of different marker genes. FEMS Microbiol. Ecol. 92, 1–12. 10.1093/femsec/fiw004 26738557

[B113] Lilley A. K. Fry J. C. Bailey M. J. Day M. J. (1996). Comparison of aerobic heterotrophic taxa isolated from four root domains of mature sugar beet (Beta vulgaris). FEMS Microbiol. Ecol. 21, 231–242. 10.1111/j.1574-6941.1996.tb00350.x

[B114] Lilley A. K. Hails R. S. Cory J. S. Bailey M. J. (1997). The dispersal and establishment of pseudomonad populations in the phyllosphere of sugar beet by phytophagous caterpillars. FEMS Microbiol. Ecol. 24, 151–157. 10.1111/j.1574-6941.1997.tb00431.x

[B115] Lim S. J. Bordenstein S. R. (2020). An introduction to phylosymbiosis. Proc. R. Soc. B Biol. Sci. 287. 10.1098/rspb.2019.2900 32126958 PMC7126058

[B116] Lindow S. E. Brandl M. T. (2003). Microbiology of the phyllosphere. Appl. Environ. Microbiol. 69, 1875–1883. 10.1128/AEM.69.4.1875-1883.2003 12676659 PMC154815

[B117] Liu H. Carvalhais L. C. Crawford M. Singh E. Dennis P. G. Pieterse C. M. J.. (2017). Inner plant values: diversity, colonization and benefits from endophytic bacteria. Front. Microbiol. 8, 1–17. 10.3389/fmicb.2017.02552 29312235 PMC5742157

[B118] Liu Y. Qi A. Khan M. F. R. (2019). Age-dependent resistance to Rhizoctonia solani in sugar beet. Plant Dis. 103, 2322–2329. 10.1094/PDIS-11-18-2001-RE 31298993

[B119] Lopisso D. T. Kühlmann V. Siebold M. (2017). Potential of soil-derived fungal biocontrol agents applied as a soil amendment and a seed coating to control Verticillium wilt of sugar beet. Biocontrol Sci. Technol. 27, 1019–1037. 10.1080/09583157.2017.1357800

[B120] Lübeck P. S. Hansen M. Sørensen J. (2000). Simultaneous detection of the establishment of seed-inoculated Pseudomonas fluorescens strain DR54 and native soil bacteria on sugar beet root surfaces using fluorescence antibody and in situ hybridization techniques. FEMS Microbiol. Ecol. 33, 11–19. 10.1016/S0168-6496(00)00038-6 10922498

[B121] Mark G. L. Dow J. M. Kiely P. D. Higgins H. Haynes J. Baysse C.. (2005). Transcriptome profiling of bacterial responses to root exudates identifies genes involved in microbe-plant interactions. Proc. Natl. Acad. Sci. U. S. A. 102, 17454–17459. 10.1073/pnas.0506407102 16301542 PMC1297666

[B122] Martin F. N. (1987). The use of Pythium oligandrum for biological control of preemergence damping-off caused by P. ultimum. Phytopathology. 77, 1013. 10.1094/Phyto-77-1013

[B123] McQuilken M. P. Whipps J. M. Cooke R. C. (1990). Control of damping-off in cress and sugar-beet by commercial seed-coating with Pythium oligandrum. Plant Pathol. 39, 452–462. 10.1111/j.1365-3059.1990.tb02521.x

[B124] Mendes R. Kruijt M. De Bruijn I. Dekkers E. Van Der Voort M. Schneider J. H. M.. (2011). Deciphering the rhizosphere microbiome for disease-suppressive bacteria. Science (80-.). 332, 1097–1100. 10.1126/science.1203980 21551032

[B125] Mendes R. Raaijmakers J. M. (2015). Cross-kingdom similarities in microbiome functions. ISME J. 9, 1905–1907. 10.1038/ismej.2015.7 25647346 PMC4542044

[B126] Milford G. F. Watson D. J. (1971). The effect of nitrogen on the growth and sugar content of sugar-beet. Ann. Bot. 35, 287–300. 10.1093/oxfordjournals.aob.a084478

[B127] Milford G. F. J. (2006). “Plant structure and crop physiology,” in Sugar Beet, Draycott A. P. (Oxford: Blackwell Publishing Ltd) p. 30–49. 10.1002/9780470751114.ch3

[B128] Monteiro F. Romeiras M. M. Batista D. Duarte M. C. (2013). Biodiversity assessment of sugar beet species and its wild relatives: Linking ecological data with new genetic approaches. Am. J. Plant Sci. 04, 21–34. 10.4236/ajps.2013.48A003

[B129] Moussa T. A. A. Rizk M. (2002). Biocontrol of sugarbeet pathogen *Fusarium solani* (Mart.) Sacc. by Streptomyces aureofaciens. Pakistan J. Biol. Sci. 5, 556–559. 10.3923/pjbs.2002.556.559

[B130] Nakayama T. Homma Y. Hashidoko Y. Mizutani J. Tahara S. (1999). Possible role of xanthobaccins produced by Stenotrophomonas sp. strain SB-K88 in suppression of sugar beet damping-off disease. Appl. Environ. Microbiol. 65, 4334–4339. 10.1128/AEM.65.10.4334-4339.1999 10508056 PMC91574

[B131] Natsagdorj O. Sakamoto H. Santiago D. M. O. Santiago C. D. Orikasa Y. Okazaki K.. (2019). Variovorax sp. has an optimum cell density to fully function as a plant growth promoter. Microorganisms. 7, 82. 10.3390/microorganisms7030082 30875976 PMC6462933

[B132] Neipp P. W. Becker J. O. (1999). Evaluation of biocontrol activity of rhizobacteria from Beta vulgaris against Heterodera schachtii. J. Nematol. 31, 54−61. 19270875 PMC2620349

[B133] Nelson E. B. (2018). The seed microbiome: origins, interactions, and impacts. Plant Soil. 422, 7–34. 10.1007/s11104-017-3289-7

[B134] Nielsen M. N. Sørensen J. Fels J. Pedersen H. C. (1998). Secondary metabolite- and endochitinase-dependent antagonism toward plant-pathogenic microfungi of Pseudomonas fluorescens isolates from sugar beet rhizosphere. Appl. Environ. Microbiol. 64, 3563–3569. 10.1128/AEM.64.10.3563-3569.1998 9758768 PMC106465

[B135] Nikolić I. Berić T. Dimkić I. Popović T. Lozo J. Fira D.. (2019). Biological control of *Pseudomonas syringae* pv. aptata on sugar beet with *Bacillus pumilus* SS-10.7 and *Bacillus amyloliquefaciens* (SS-12.6 and SS-38.4) strains. J. Appl. Microbiol. 126, 165–176. 10.1111/jam.14070 30117660

[B136] Nogales A. Nobre T. Valadas V. Ragonezi C. Döring M. Polidoros A.. (2016). Can functional hologenomics aid tackling current challenges in plant breeding? Brief. Funct. Genomics 15, 288–297. 10.1093/bfgp/elv030 26293603

[B137] Novinscak A. Filion M. (2011). Effect of soil clay content on RNA isolation and on detection and quantification of bacterial gene transcripts in soil by quantitative reverse transcription-PCR. Appl. Environ. Microbiol. 77, 6249–6252. 10.1128/AEM.00055-11 21724880 PMC3165432

[B138] Ogata N. Takahashi H. Taguchi K. Okazaki K. Nakatsuka K. (2006). Change in the amount of amino nitrogen in crown root of sugar beet (Beta vulgaris L.) infected with Rhizoctonia solani. Japanese J. Crop Sci. 74, 357–363. 10.1626/jcs.74.357

[B139] Okazaki K. Iino T. Kuroda Y. Taguchi K. Takahashi H. Ohwada T.. (2014). An assessment of the diversity of culturable bacteria from main root of sugar beet. Microbes Environ. 29, 220–223. 10.1264/jsme2.ME13182 24789987 PMC4103529

[B140] Okazaki K. Tsurumaru H. Hashimoto M. Takahashi H. Okubo T. Ohwada T.. (2021). Community analysis-based screening of plant growth-promoting bacteria for sugar beet. Microbes Environ. 36, 1–11. 10.1264/jsme2.ME20137 33907063 PMC8209457

[B141] Orozco-Mosqueda M. del C. Rocha-Granados M. del C. Glick B. R. Santoyo G. (2018). Microbiome engineering to improve biocontrol and plant growth-promoting mechanisms. Microbiol. Res. 208, 25–31. 10.1016/j.micres.2018.01.005 29551209

[B142] Osburn R. M. (1989). Dynamics of sugar beet seed colonization by Pythium ultimum and Pseudomonas species: effects on seed rot and damping-off. Phytopathology. 79, 709. 10.1094/Phyto-79-709

[B143] Ottesen A. R. González Peña A. White J. R. Pettengill J. B. Li C. Allard S.. (2013). Baseline survey of the anatomical microbial ecology of an important food plant: *Solanum lycopersicum* (tomato). *BMC Microbiol*. 13, 114. 10.1186/1471-2180-13-114 23705801 PMC3680157

[B144] Owen F. W. Carsner E. Stout M. (1940). Photothermal induction of flowering in sugar beet. Am. Soc. Sugar Beet Technol. 61, 101–124.

[B145] Palumbo J. D. Yuen G. Y. Jochum C. C. Tatum K. Kobayashi D. Y. (2005). Mutagenesis of β-1,3-glucanase genes in *Lysobacter enzymogenes* strain C3 results in reduced biological control activity toward Bipolaris leaf spot of tall fescue and Pythium damping-off of sugar beet. Phytopathology. 95, 701–707. 10.1094/PHYTO-95-0701 18943787

[B146] Panella L. Campbell L. G. Eujayl I. A. Lewellen R. T. McGrath J. M. (2015). USDA-ARS sugarbeet releases and breeding over the past 20 years. J. Sugarbeet Res. 52, 1–2. 10.5274/jsbr.52.3.40

[B147] Panella L. Lewellen R. T. (2007). Broadening the genetic base of sugar beet: introgression from wild relatives. Euphytica. 154, 383–400. 10.1007/s10681-006-9209-1

[B148] Pantigoso H. A. Newberger D. Vivanco J. M. (2022). The rhizosphere microbiome: plant–microbial interactions for resource acquisition. J. Appl. Microbiol. 133, 2864–2876. 10.1111/jam.15686 36648151 PMC9796772

[B149] Pérez-Jaramillo J. E. Carrión V. J. Bosse M. Ferrão L. F. V. De Hollander M. Garcia A. A. F.. (2017). Linking rhizosphere microbiome composition of wild and domesticated *Phaseolus vulgaris* to genotypic and root phenotypic traits. ISME J. 11, 2244–2257. 10.1038/ismej.2017.85 28585939 PMC5607367

[B150] Pérez-Jaramillo J. E. Carrión V. J. de Hollander M. Raaijmakers J. M. (2018). The wild side of plant microbiomes. Microbiome. 6, 4–9. 10.1186/s40168-018-0519-z 30115122 PMC6097318

[B151] Pervaiz Z. H. Iqbal J. Zhang Q. Chen D. Wei H. Saleem M. (2020). Continuous cropping alters multiple biotic and abiotic indicators of soil health. Soil Syst. 4, 59. 10.3390/soilsystems4040059

[B152] Philippot L. Raaijmakers J. M. Lemanceau P. van der Putten W. H. (2013). Going back to the roots: the microbial ecology of the rhizosphere. Nat. Rev. Microbiol. 11, 789–799. 10.1038/nrmicro3109 24056930

[B153] Piernik A. Hrynkiewicz K. Wojciechowska A. Szymańska S. Lis M. I. Muscolo A. (2017). Effect of halotolerant endophytic bacteria isolated from Salicornia europaea L. on the growth of fodder beet (*Beta vulgaris* L.) under salt stress. Arch. Agron. Soil Sci. 63, 1404–1418. 10.1080/03650340.2017.1286329

[B154] Pigoleva S. V. Zakharchenko N. S. Pigolev A. V. Trotsenko Y. A. Buryanov Y. I. (2009). The influence of colonizing methylobacteria on morphogenesis and resistance of sugar beet and white cabbage plants to Erwinia carotovora. Appl. Biochem. Microbiol. 45, 604–609. 10.1134/S0003683809060052 20067151

[B155] Postma J. Schilder M. T. (2015). Enhancement of soil suppressiveness against Rhizoctonia solani in sugar beet by organic amendments. Appl. Soil Ecol. 94, 72–79. 10.1016/j.apsoil.2015.05.00233771785

[B156] Pusenkova L. I. Il'yasova E. Y. Lastochkina O. V. Maksimov I. V. Leonova S. A. (2016). Changes in the species composition of the rhizosphere and phyllosphere of sugar beet under the impact of biological preparations based on endophytic bacteria and their metabolites. Eurasian Soil Sci. 49, 1136–1144. 10.1134/S1064229316100112

[B157] Raaijmakers J. M. Mazzola M. (2016). Soil immune responses. Science (80-.). 352, 1392–1393. 10.1126/science.aaf3252 27313024

[B158] Rainey P. B. Bailey M. J. Thompson I. P. (1994). Phenotypic and genotypic diversity of fluorescent pseudomonads isolated from field-grown sugar beet. Microbiology. 140, 2315–2331. 10.1099/13500872-140-9-2315 7952185

[B159] Russo A. Basaglia M. Tola E. Casella S. (2001). Survival, root colonisation and biocontrol capacities of *Pseudomonas fluorescens* F113 LacZY in dry alginate microbeads. J. Ind. Microbiol. Biotechnol. 27, 337–342. 10.1038/sj.jim.7000154 11773997

[B160] Rybakova D. Mancinelli R. Wikström M. Birch-Jensen A. S. Postma J. Ehlers R. U.. (2017). The structure of the *Brassica napus* seed microbiome is cultivar-dependent and affects the interactions of symbionts and pathogens. Microbiome 5, 104. 10.1186/s40168-017-0310-6 28859671 PMC5580328

[B161] Sadeghi A. Hesan A. R. Askari H. Qomi D. N. Farsi M. Hervan E. M. (2009). Biocontrol of Rhizoctonia solani damping-off of sugar beet with native Streptomyces strains under field conditions. Biocontrol Sci. Technol. 19, 985–991. 10.1080/09583150902912665

[B162] Saftner R. A. Daie J. Wyse R. E. (1983). Sucrose uptake and compartmentation in sugar beet taproot tissue. Plant Physiol. 72, 1–6. 10.1104/pp.72.1.1 16662941 PMC1066159

[B163] Samadi A. (2012). Impact of continuous sugar beet cropping on potassium quantity-intensity parameters in calcareous soils. J. Plant Nutr. 35, 1154–1167. 10.1080/01904167.2012.676128

[B164] Sasse J. Martinoia E. Northen T. (2018). Feed your friends: do plant exudates shape the root microbiome? Trends Plant Sci. 23, 25–41. 10.1016/j.tplants.2017.09.003 29050989

[B165] Scholten O. E. Lange W. (2000). Breeding for resistance to rhizomania in sugar beet: a review. Euphytica. 112, 219–231. 10.1023/A:100398800316519161359

[B166] Shahzad R. Khan A. L. Bilal S. Asaf S. Lee I. J. (2018). What is there in seeds? Vertically transmitted endophytic resources for sustainable improvement in plant growth. Front. Plant Sci. 9, 1–10. 10.3389/fpls.2018.00024 29410675 PMC5787091

[B167] Shi Y. Lou K. Li C. (2009a). Isolation, quantity distribution and characterization of endophytic microorganisms within sugar beet. African J. Biotechnol. 8, 835–840.

[B168] Shi Y. Lou K. Li C. (2009b). Promotion of plant growth by phytohormone-producing endophytic microbes of sugar beet. Biol. Fertil. Soils. 45, 645–653. 10.1007/s00374-009-0376-9

[B169] Shi Y. Lou K. Li C. (2011). Growth promotion effects of the endophyte Acinetobacter johnsonii strain 3-1 on sugar beet. Symbiosis. 54, 159–166. 10.1007/s13199-011-0139-x

[B170] Shi Y. Yang H. Zhang T. Sun J. Lou K. (2014). Illumina-based analysis of endophytic bacterial diversity and space-time dynamics in sugar beet on the north slope of Tianshan mountain. Appl. Microbiol. Biotechnol. 98, 6375–6385. 10.1007/s00253-014-5720-9 24752839

[B171] Shi Y. W. Li C. Yang H. M. Zhang T. Gao Y. Chu M.. (2017). Colonization study of gfp-tagged Achromobacter marplatensis strain in sugar beet. J. Microbiol. 55, 267–272. 10.1007/s12275-017-6371-1 28124776

[B172] Shi Y. W. Li C. Yang H. M. Zhang T. Gao Y. Zeng J.. (2016). Endophytic fungal diversity and space-time dynamics in sugar beet. Eur. J. Soil Biol. 77, 77–85. 10.1016/j.ejsobi.2016.09.005

[B173] Shi Y. W. TaPa M. S. Li C. Yang H. M. Zhang T. Gao Y.. (2015). Diversity and space–time dynamics of endophytic archaea from sugar beet in the north slope of Tianshan Mountain revealed by 454 pyrosequencing and T-RFLP. World J. Microbiol. Biotechnol. 31, 1031–1039. 10.1007/s11274-015-1853-y 25862354

[B174] Shimizu Y. Sagiya D. Matsui M. Fukui R. (2018). Zonal soil amendment with simple sugars to elevate soil C/N ratios as an alternative disease management strategy for Rhizoctonia damping-off of sugar beet. Plant Dis. 102, 1434–1444. 10.1094/PDIS-09-16-1279-RE 30673559

[B175] Smirnova I. Sadanov A. (2019). Application of agriculturally important microorganisms for biocontrol of root rot infection of sugar beet. Arch. Phytopathol. Plant Prot. 52, 698–713. 10.1080/03235408.2019.1588195

[B176] Spanner R. Neubauer J. Heick T. M. Grusak M. A. Hamilton O. Rivera-Varas V.. (2021). Seedborne *Cercospora beticola* can initiate Cercospora leaf spot from sugar beet (Beta vulgaris) fruit tissue. Phytopathology. 112, 1016–1028. 10.1094/PHYTO-03-21-0113-R 34844416

[B177] Steinkellner S. Lendzemo V. Langer I. Schweiger P. Khaosaad T. Toussaint J. P.. (2007). Flavonoids and strigolactones in root exudates as signals in symbiotic and pathogenic plant-fungus interactions. Molecules. 12, 1290–1306. 10.3390/12071290 17909485 PMC6149470

[B178] Stevanato P. Chiodi C. Broccanello C. Concheri G. (2019). Sustainability of the sugar beet crop. Sugar Tech. 21, 703–716. 10.1007/s12355-019-00734-9

[B179] Stevanato P. Squartini A. Concheri G. Saccomani M. (2016). Sugar beet yield and processing quality in relation to nitrogen content and microbiological diversity of deep soil layer. Sugar Tech 18, 67–74. 10.1007/s12355-014-0365-7

[B180] Stevanato P. Zavalloni C. Marchetti R. Bertaggia M. Saccomani M. McGrath J. M.. (2010). Relationship between subsoil nitrogen availability and sugarbeet processing quality. Agron. J. 102, 17–22. 10.2134/agronj2009.0041

[B181] Takenaka S. Nakamura Y. Kono T. Sekiguchi H. Masunaka A. Takahashi H. (2006). Novel elicitin-like proteins isolated from the cell wall of the biocontrol agent Pythium oligandrum induce defence-related genes in sugar beet. Mol. Plant Pathol. 7, 325–339. 10.1111/j.1364-3703.2006.00340.x 20507450

[B182] Takenaka S. Nishio Z. Nakamura Y. (2003). Induction of defense reactions in sugar beet and wheat by treatment with cell wall protein fractions from the mycoparasite *Pythium oligandrum*. Phytopathology. 93, 1228–1232. 10.1094/PHYTO.2003.93.10.1228 18944321

[B183] Thompson I. P. Bailey M. J. Ellis R. J. Lilley A. K. McCormack P. J. Purdy K. J.. (1995a). Short-term community dynamics in the phyllosphere microbiology of field-grown sugar beet. FEMS Microbiol. Ecol. 16, 205–211. 10.1111/j.1574-6941.1995.tb00284.x

[B184] Thompson I. P. Bailey M. J. Fenlon J. S. Fermor T. R. Lilley A. K. Lynch J. M.. (1993). Quantitative and qualitative seasonal changes in the microbial community from the phyllosphere of sugar beet (Beta vulgaris). Plant Soil. 150, 177–191. 10.1007/BF00013015

[B185] Thompson I. P. Ellis R. J. Bailey M. J. (1995b). Autecology of a genetically modified fluorescent pseudomonad on sugar beet. FEMS Microbiol. Ecol. 17, 1–13. 10.1111/j.1574-6941.1995.tb00122.x

[B186] Thrane C. Harder Nielsen T. Neiendam Nielsen M. Sørensen J. Olsson S. (2000). Viscosinamide-producing *Pseudomonas fluorescens* DR54 exerts a biocontrol effect on Pythium ultimum in sugar beet rhizosphere. FEMS Microbiol. Ecol. 33, 139–146. 10.1111/j.1574-6941.2000.tb00736.x 10967213

[B187] Truyens S. Weyens N. Cuypers A. Vangronsveld J. (2013). Changes in the population of seed bacteria of transgenerationally Cd-exposed Arabidopsis thaliana. Plant Biol. 15, 971–981. 10.1111/j.1438-8677.2012.00711.x 23252960

[B188] Truyens S. Weyens N. Cuypers A. Vangronsveld J. (2015). Bacterial seed endophytes: Genera, vertical transmission and interaction with plants. Environ. Microbiol. Rep. 7, 40–50. 10.1111/1758-2229.1218131798570

[B189] Tsurumaru H. Okubo T. Okazaki K. Hashimoto M. Kakizaki K. Hanzawa E.. (2015). Metagenomic analysis of the bacterial community associated with the taproot of sugar beet. Microbes Environ. 30, 63–69. 10.1264/jsme2.ME14109 25740621 PMC4356465

[B190] van der Voort M. Kempenaar M. van Driel M. Raaijmakers J. M. Mendes R. (2016). Impact of soil heat on reassembly of bacterial communities in the rhizosphere microbiome and plant disease suppression. Ecol. Lett. 19, 375–382. 10.1111/ele.12567 26833547

[B191] Vida C. de Vicente A. Cazorla F. M. (2020). The role of organic amendments to soil for crop protection: induction of suppression of soilborne pathogens. Ann. Appl. Biol. 176, 1–15. 10.1111/aab.12555

[B192] Wassermann B. Abdelfattah A. Wicaksono W. A. Kusstatscher P. Müller H. Cernava T.. (2022). The Brassica napus seed microbiota is cultivar-specific and transmitted via paternal breeding lines. Microb. Biotechnol. 15, 2379–2390. 10.1111/1751-7915.14077 35593114 PMC9437892

[B193] Wassermann B. Cernava T. Müller H. Berg C. Berg G. (2019a). Seeds of native alpine plants host unique microbial communities embedded in cross-kingdom networks. Microbiome. 7, 108. 10.1186/s40168-019-0723-5 31340847 PMC6651914

[B194] Wassermann B. Müller H. Berg G. (2019b). An apple a day: which bacteria do we eat with organic and conventional apples? Front. Microbiol. 10, 1–13. 10.3389/fmicb.2019.01629 31396172 PMC6667679

[B195] Weller D. M. Raaijmakers J. M. Gardener B. B. M. Thomashow L. S. (2002). Microbial populations responsible for specific soil suppressiveness to plant pathogens. Annu. Rev. Phytopathol. 40, 309–348. 10.1146/annurev.phyto.40.030402.110010 12147763

[B196] Whitney E. D. (1989). Identification, distribution, and testing for resistance to rhizomania in Beta maritima. Plant Dis. 73, 287–290. 10.1094/PD-73-0287

[B197] Williams G. E. Asher M. J. C. (1996). Selection of rhizobacteria for the control of *Pythium ultimum* and *Aphanomyces cochlioides* on sugar-beet seedlings. Crop Prot. 15, 479–486. 10.1016/0261-2194(96)00014-2

[B198] Windels C. E. Jacobsen B. J. Harveson R. M. (2009). “Rhizoctonia root and crown rot,” in Compendium of beet diseases and pests, eds. Harveson R. M. Hanson L. E. Hein G. L. (St. Paul, MN: American Phytopathological Society Press), 33–36.

[B199] Wolfgang A. Zachow C. Müller H. Grand A. Temme N. Tilcher R.. (2020). Understanding the impact of cultivar, seed origin, and substrate on bacterial diversity of the sugar beet rhizosphere and suppression of soil-borne pathogens. Front. Plant Sci. 11, 1–15. 10.3389/fpls.2020.560869 33101330 PMC7554574

[B200] Wyse R. (1979). Parameters controlling sucrose content and yield of sugarbeet roots. 20th Gen. Meet. Am. Soc. Sugarbeet Technol. 20, 368–385. 10.5274/jsbr.20.4.368

[B201] Yilmaz N. D. K. Tunali B. (2010). Evaluation of Trichoderma spp. from central and northern regions of Turkey for suppression of *Polymyxa betae* as a vector of rhizomania disease. Arch. Phytopathol. Plant Prot. 43, 1534–1542. 10.1080/03235400902927154

[B202] Zachow C. Fatehi J. Cardinale M. Tilcher R. Berg G. (2010). Strain-specific colonization pattern of Rhizoctonia antagonists in the root system of sugar beet. FEMS Microbiol. Ecol. 74, 124–135. 10.1111/j.1574-6941.2010.00930.x 20618857

[B203] Zachow C. Jahanshah G. Bruijn I. De Song C. Ianni F. Gerhardt H.. (2015). The novel lipopeptide poaeamide of the endophyte Pseudomonas poae RE ^*^ 1-1-14 is involved in pathogen suppression and root colonization. Mol. Plant-Microbe Interact. 28, 800–810. 10.1094/MPMI-12-14-0406-R 25761208

[B204] Zachow C. Müller H. Tilcher R. Berg G. (2014). Differences between the rhizosphere microbiome of Beta vulgaris ssp. maritima - ancestor of all beet crops–and modern sugar beets. Front. Microbiol. 5, 1–13. 10.3389/fmicb.2014.00415 25206350 PMC4144093

[B205] Zachow C. Müller H. Tilcher R. Donat C. Berg G. (2013). Catch the best: Novel screening strategy to select stress protecting agents for crop plants. Agronomy. 3, 794–815. 10.3390/agronomy3040794

[B206] Zachow C. Tilcher R. Berg G. (2008). Sugar beet-associated bacterial and fungal communities show a high indigenous antagonistic potential against plant pathogens. Microb. Ecol. 55, 119–129. 10.1007/s00248-007-9257-7 18060449

[B207] Zilber-Rosenberg I. Rosenberg E. (2008). Role of microorganisms in the evolution of animals and plants: the hologenome theory of evolution. FEMS Microbiol. Rev. 32, 723–735. 10.1111/j.1574-6976.2008.00123.x 18549407

